# COVID-19 pandemic effects on health worker’s mental health: Systematic review and meta-analysis

**DOI:** 10.1192/j.eurpsy.2022.1

**Published:** 2022-01-21

**Authors:** Claudia Aymerich, Borja Pedruzo, Jose Luís Pérez, Maria Laborda, Jon Herrero, Jorge Blanco, Gonzalo Mancebo, Lucía Andrés, Olatz Estévez, Maitane Fernandez, Gonzalo Salazar de Pablo, Ana Catalan, Miguel Ángel González-Torres

**Affiliations:** 1 Psychiatry Department, Basurto University Hospital, Bilbao, Spain; 2 Department of Psychosis Studies, Early Psychosis: Interventions and Clinical-detection (EPIC) Lab, Institute of Psychiatry, Psychology & Neuroscience, King’s College London, London, United Kingdom; 3 Department of Child and Adolescent Psychiatry, Institute of Psychiatry and Mental Health, Hospital General Universitario Gregorio Marañón, Madrid, Spain; 4 Biocruces Bizkaia Health Research Institute, Barakaldo, Spain; 5 Neuroscience Department, University of the Basque Country, UPV/EHU, Leioa, Spain; 6 Department of Psychosis Studies, Institute of Psychiatry, Psychology & Neuroscience, King’s College London, London, United Kingdom

**Keywords:** Coronavirus, COVID-19, healthcare workers, mental health

## Abstract

**Background:**

Healthcare workers (HCWs) exposed to coronavirus 19 (COVID-19) are at high risk of developing mental health concerns across several domains. The aim of this study is to determine the updated, global frequency of these outcomes.

**Methods:**

A multistep literature search was performed from database inception until March 1, 2021. PRISMA/MOOSE-compliant systematic review and PROSPERO protocol were used to identify studies reporting on depression, anxiety, acute stress, post-traumatic symptoms, insomnia, and burnout in HCWs exposed to COVID-19. A quantitative meta-analysis with random effects was conducted to analyze the proportion rate of the mental health disorders. Sensitivity analyses were performed to investigate the effect of the different continents and scales. Meta-regression analyses were conducted to examine the effect of gender, age, and work position.

**Results:**

239 articles were included (*n* = 271,319 HCWs, mean age = 36.08 ± 8.33 (66.99% female). 33% HCWs exposed to COVID-19 reported depressive symptoms (95% confidence intervals [CI] = 28–38%), 42% anxiety features (95% CI = 35–48), 40% acute stress (95% CI = 32–47), 32% post-traumatic symptoms (95% CI = 26–37%), 42% insomnia (95% CI = 36–48), 37% burnout (95% CI = 31–42). Sensitivity analyses did not show statistically significant differences. Meta-regressions found a statistically significant lower prevalence of post-traumatic symptoms in Asia.

**Conclusions:**

HCWs exposed to COVID-19 were found to have a significant prevalence of mental health concerns in all domains analyzed. The effects of COVID-19 on HCWs’ mental health could be underestimated and the future consequences dismissed.

## Introduction

On December 31, 2019, the WHO warned of the first cases of pneumonia caused by a new coronavirus in the city of Wuhan [1]. As of September 1, 2021, the disease caused by this virus (the COVID-19) has infected more than 215 million people worldwide and caused 4.5 million deaths, thus being considered a global pandemic [2].

Large outbreaks such as the one caused by COVID-19 place healthcare workers (HCWs) in a position of particular vulnerability [3]. HCWs are not only one of the groups most at risk of being infected by COVID-19 [4], but they are exposed to a huge workload [5], the absence of adequate protective equipment [6] and the extensive media coverage [7,8]. Routine clinical practice has been significantly changed, and many professionals have been removed from their usual workplace and redirected to higher-risk frontline works while also having to adhere to continuously changing guidelines [9].

The literature published during the SARS and MERS pandemics more than a decade ago suggests that HCWs present, due to all the above, an increased risk of suffering adverse mental health effects in pandemic situations, including anxiety, depression, and post-traumatic stress symptoms [10–14]. In line with these results, recently numerous scientific articles have been published on this subject. Most of these studies and reviews, however, focus on one or few mental health domains or offer results from very specific populations, either in terms of geographical origin (mainly from mainland China) or professional category and medical specialty [15–18].

No updated meta-analyses analyze the effects of the COVID-19 pandemic on the different domains of mental health impact in HCWs worldwide, including depression, anxiety, burnout, acute stress, post-traumatic symptoms, and insomnia. Therefore, the aim of this study is to synthesize the available scientific evidence about the state of mental health of HCWs during the COVID-19 pandemic.

## Methods

This study protocol was registered on PROSPERO (registration number: CRD42021247610). The study was conducted in accordance with “Preferred Reporting Items for Systematic Reviews and Meta-Analyses” (PRISMA) [19], (Supplementary Table S1) and “Meta-analyses of Observational Studies in Epidemiology” (MOOSE) checklist [20] (Supplementary Table S2), following “EQUATOR Reporting Guidelines” [21].

### Search strategy and selection criteria

A systematic literature search was carried out by two independent researchers (C.A. and B.P.). Web of Science database (Clarivate Analytics) was searched, incorporating the Web of Science Core Collection, the BIOSIS Citation Index, the KCI-Korean Journal Database, MEDLINE®, the Russian Science Citation Index, and the SciELO Citation Index as well as Cochrane Central Register of Reviews, and Ovid/PsycINFO databases, from inception until March 1, 2021.

The following keywords were used: “CoV-19” OR “SARS-CoV-2” OR “2019 nCoV” OR “2019nCoV” OR “2019 novel coronavirus” OR “new coronavirus” OR “novel coronavirus” OR “SARS CoV-2” OR “Wuhan coronavirus” OR “COVID 19” OR “2019-nCoV” AND “professionals” OR “worker*” OR “doctor*” OR “nurse*” OR “occupation*” OR “employee*” OR “healthcare provider*” OR “healthcare worker*” OR “healthcare employee*” OR “personnel” OR “emergency worker” OR “paramedic*”.

Articles identified were first screened as abstracts, and after the exclusion of those which did not meet the inclusion criteria, the full texts of the remaining articles were assessed for eligibility and inclusion.

Inclusion criteria for the systematic review and meta-analysis were (a) individual studies with original data, (b) focusing on HCWs exposed to COVID-19 (defined as HCWs who have been working during COVID-19 pandemic tending to patients potentially infected with SARS-COV-2, but not necessarily limited toHCWs working in frontline units), (c) reporting meta-analyzable proportions about mental health outcomes included in at least one of the following categories: anxiety, depression, acute stress/distress, post-traumatic symptoms, burnout, and sleep disturbances, (d) using validated, structured, evaluation scales, (e) nonoverlapping samples (overlap was determined by looking at the inclusion dates, type of population and country in which the study was carried out, and the study with the largest sample was then selected), (f) sample size ≥50 participants, and (g) written in English. Exclusion criteria were (a) reviews, clinical cases, study protocols or qualitative studies, conferential proceedings, letters, and commentaries, (b) reporting outcomes on populations other than HCWs, including the general population, medical and nursing students, dentists, and podologists.

### Data extraction

Three researchers (J.L.P., M.L., and J.H.) independently extracted data from all the included studies. The three databases were then cross-checked, and discrepancies were resolved through consensus under the supervision of a senior researcher (A.C.). A summary of selected variables included: first author and year of publication, country and city, HCW category involved, sample size, age (mean ± standard deviation [SD]), sex (% female), mental health domain studied, evaluation tool used, quality assessment (see below), and key findings.

### Risk of bias (quality) assessment

Risk of bias was assessed using a modified version of the Newcastle–Ottawa Scale (NOS) for assessing the quality of nonrandomized studies due to the heterogeneity expected in the included studies [22] (Supplementary Methods S1).

### Strategy for data synthesis

First, we provided a systematic synthesis (Supplementary Table S4) of the findings from the included studies structured around the selected six mental health outcomes: anxiety, depression, acute stress/distress, post-traumatic symptoms, burnout, and sleep disturbances. Second, we performed meta-analyses using, as primary effect size, the proportion (% and standard error [SE], when available) of mental health outcomes in HCWs exposed to COVID-19.

Meta-regressions were performed to determine the effect of the (a) sex, (b) age, and (c) NOS score on the mental health domains. Sensitivity analyses were conducted to estimate the association between the mental health domains and (a) continent of the study, (b) type of mental health worker (doctor, nurse, or multi-professional), and (c) used scale.

Heterogeneity among studies was assessed using the Q statistic, with the proportion of the total variability in effect size estimates evaluated using the I2 index (with an I2 > 50% representing significant heterogeneity) [23]. Publication biases were assessed for the proportion of remission or recovery by inspecting funnel plots and assessing Egger’s test [24].

All analyzes were conducted using STATA version 17 [25]. The significance level was set at a *p* < 0.05, two-sided.

## Results

**Figure 1.**
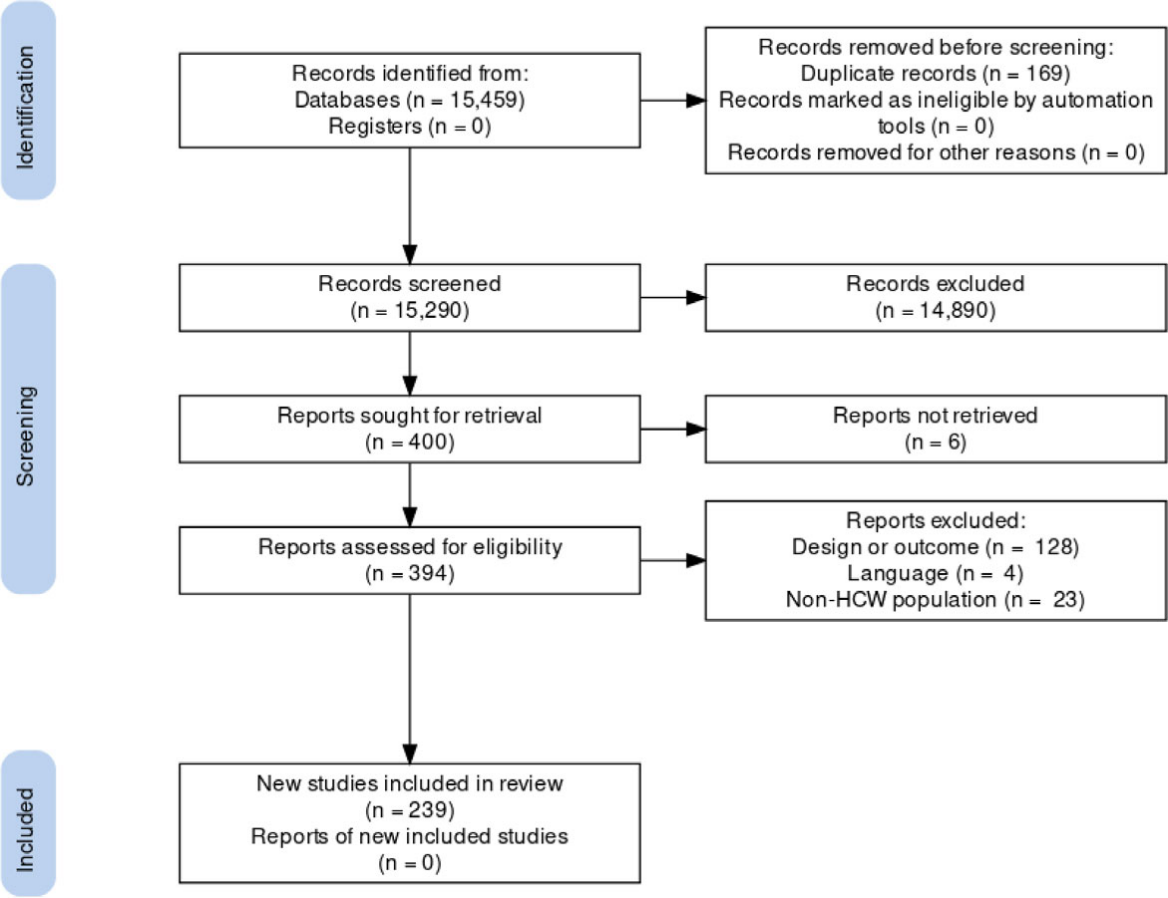
PRISMA 2009 flow diagram [26].

The literature search yielded 15,459 citations through electronic database, which were screened for eligibility; 394 articles were assessed in full text, and 155 were excluded (reasons for exclusion are detailed in Supplementary Table S3). The final database for the systematic review and meta-analysis included 239 studies (Figure 1).

One hundred and sixty studies (66.95%) focused on depression, 179 (74.90%) on anxiety, 57 (23.85%) on acute stress/distress, 55 (23.01%) on sleep problems, 39 (16.32%) on post-traumatic symptoms, and 24 (10.04%) on burnout. The full sample includes 271,319 HCWs, including articles with sample sizes ranging from 54 to 21,199 HCWs. The mean age of the sample was 36.08 years, ranging from 21 to 55.13 years (SD = 8.33). 66.99% were female. Studies included HCWs from 50 countries in five continents: 150 (62.76%) from Asia, 55 (23.01%) from Europe, 20 (8.37%) from America, 11 (4.60%) from Africa, and 2 (0.84%) from Oceania; there was also one multicontinental study [27].

### Depression

Depression prevalence was reported in 160 studies, including a total sample of 210,762 participants. Multiple evaluation scales were used, including Patient Health Questionnaire-9 (PHQ-9) [28], PHQ-2 [29], PHQ-4 [30], Zung Self-Rating Depression Scale (SDS) [31], Hospital Anxiety and Depression Scale (HADS) [32], Beck Depression Inventory (BDI) [33], Center for Epidemiologic Studies Depression Scale (CES-D) [34], and Depression, Anxiety, and Stress Scale-21 (DASS-21) [35]. The pooled prevalence of depression was 0.33 (95% confidence intervals [CI] 0.28–0.38). Prevalence varied widely depending on the scale used, from 0.53 with PHQ-2/4 to 0.26 with CES-D. Detailed results of depression and the other mental health domains are displayed in Table 1. Sensitivity analyses and meta-regressions revealed no statistically significant differences regarding age, gender, NOS score, or continent.

**Table 1.** Prevalence of mental health impacts across each of the domains and scales studied.

Scale	No. Studies	Sample size	Proportion	95% CI	*z* Score	*p*	*I* ^2^ (%)
**Depression**	160	210,762	0.33	0.28–0.38	13.36	0.00	99.95
PHQ9	74	115,185	0.33	0.28–0.38	11.98	0.00	99.85
SDS	12	26,207	0.38	0.10–0.65	2.69	0.01	99.97
HADS	23	12,984	0.34	0.27–0.42	9.40	0.00	98.85
BDI	5	2,325	0.35	0.22–0.47	5.40	0.00	96.54
CES-D	3	16,118	0.30	0.24–0.36	9.78	0.00	n.a.
DASS-21	36	23,497	0.33	0.24–0.42	7.42	0.00	99.87
PHQ2/PHQ4	4	4,005	0.25	0.19–0.30	8.78	0.00	91.56
**Anxiety**	179	206,513	0.42	0.35–0.48	12.35	0.00	99.94
GAD-7	87	123,895	0.42	0.35–0.48	12.72	0.00	99.87
DASS-21	32	22,698	0.37	0.29–0.44	9.59	0.00	99.22
HADS	22	14,515	0.44	0.39–0.50	15.41	0.00	96.25
SAS	16	32,000	0.35	0.08–0.62	2.52	0.00	99.98
BAI	4	1,569	0.34	0.11–0.58	2.94	0.00	98.49
STAI-S	10	5,875	0.68	0.48–0.88	6.68	0.00	99.77
PHQ-4	4	4,005	0.37	0.23–0.51	5.20	0.00	98.52
CAS	4	2,018	0.47	0.27–0.67	4.66	0.00	n.a.
**Acute stress**	57	48,042	0.40	0.32–0.47	10.12	0.00	99.87
SASRQ	4	12,365	0.33	0.10–0.55	2.83	0.00	99.73
DASS-21	32	22,561	0.26	0.21–0.32	9.37	0.00	99.42
PSS	21	13,116	0.62	0.50–0.73	10.35	0.00	99.67
**Post-trauma**	39	58,995	0.32	0.26–0.37	11.01	0.00	99.69
IES-R	23	24,225	0.38	0.29–0.47	8.33	0.00	99.70
PCL-C	12	26,057	0.20	0.12–0.28	4.94	0.00	99.53
PC-PTSD	4	8,713	0.32	0.21–0.43	5.77	0.00	99.07
**Insomnia**	55	37,068	0.42	0.36–0.48	12.92	0.00	99.59
ISI	27	23,366	0.39	0.30–0.47	8.96	0.00	99.66
SQS	3	920	0.28	0.02–0.54	2.11	0.04	n.a.
AIS	6	4,079	0.42	0.33–0.51	9.42	0.00	96.66
PSQI	19	8,703	0.49	0.37–0.61	8.26	0.00	99.29
**Burnout**	25	30,873	0.37	0.31–0.42	12.62	0.00	99.81
MBI-EE	19	26,776	0.39	0.30–0.48	8.90	0.00	99.50
MBI-DP	19	26,776	0.34	0.22–0.46	5.59	0.00	99.77
MBI-RPA	19	26,776	0.36	0.28–0.45	8.54	0.00	99.38
CBI	3	3,238	0.53	0.52–0.54	59.85	0.00	n.a.
Mini-Z	3	859	0.22	0.14–0.30	5.31	0.00	n.a.

Abbreviations: AIS, Athens Insomnia Scale; BAI, Beck Anxiety Inventory; BDI, Beck Depression Inventory; CAS, Coronavirus Anxiety Scale; CBI, Copenhagen Burnout Inventory; CES-D, Center for Epidemiologic Studies Depression Scale; DASS-21, Depression, Anxiety, and Stress Scale-21; GAD-7, Generalized Anxiety Disorder-7; HADS, Hospital Anxiety and Depression Scale; IES-R, Impact of Event Scale—Revised; ISI, Insomnia Severity Index; MBI-DP, Maslach Burnout Inventory—depersonalization; MBI-EE, Maslach Burnout Inventory—emotional exhaustion; MBI-RPA, Maslach Burnout Inventory—reduced personal accomplishment; Mini-Z, Mini-Z Burnout Survey; n.a., not applicable; PC-PTSD, Primary Care—Post Traumatic Stress Disorder Scale; PCL-C, Post Traumatic Stress Disorder Checklist—Civilian Version; PHQ, Patient Health Questionnaire; PSQI, Pittsburgh Sleep Quality Index; SAS, Zung Self-Rating Anxiety Scale; SASRQ, Stanford Acute Stress Reaction Questionnaire; SDS, Zung Self-Rating Depression Scale; SQS, Sleep Quality Scale; STAI-S, State-–Trait Anxiety Inventory—State Subscale.

### Anxiety

Anxiety prevalence was reported in 179 studies, including a total sample of 206,513 participants. Multiple evaluation scales were used, including Generalized Anxiety Disorder-7 (GAD-7) [36], DASS-21 [35], HADS [32], Zung Self-Rating Anxiety Scale (SAS) [37], Beck Anxiety Inventory (BAI) [38], State–Trait Anxiety Inventory—State Subscale (STAI-S) [39], PHQ-4 [30], and Coronavirus Anxiety Scale (CAS) [40]. The pooled prevalence of anxiety was 0.42 (95% CI 0.35–0.48). Again, prevalence varied substantially depending on the scale used, from 0.34 with BAI to 0.68 with STAI-S. Sensitivity analyses and meta-regressions did not show statistically significant differences regarding age, gender, NOS score, or continent.

### Acute stress

Acute stress prevalence was reported in 57 studies, including a total sample of 48,042 participants. Multiple evaluation scales were used, including Stanford Acute Stress Reaction Questionnaire (SASRQ) [41], DASS-21 [35], and Perceived Stress Scale (PSS) [42]. The pooled prevalence of acute stress was 0.40 (95% CI 0.32–0.47). Prevalence varied from 0.26 as measured with DASS-21 to 0.62 with PSS. Again, sensitivity analyses and meta-regressions revealed no statistically significant differences regarding age, gender, NOS score, continent, or professional category.

### Insomnia

Insomnia prevalence was reported in 55 studies, including a total sample of 37,068 participants. Multiple evaluation scales were used, including Insomnia Severity Index (ISI) [43], Sleep Quality Scale (SQS) [44], Athens Insomnia Scale (AIS) [45], and Pittsburgh Sleep Quality Index (PSQI) [46]. The pooled prevalence of insomnia was 0.42 (95% CI 0.36–0.48). Sensitivity analyses and meta-regressions revealed no statistically significant differences regarding age, gender, NOS score, or continent.

### Post-traumatic symptoms

Relevant post-traumatic symptoms prevalence was reported in 39 studies, including a total sample of 58,995 participants. Multiple evaluation scales were used, including Impact of Event Scale—Revised (IES-R) [47], Post Traumatic Stress Disorder Checklist—Civilian Version (PCL-C) [48], and Primary Care—Post Traumatic Stress Disorder Scale (PC-PTSD) [49]. The pooled prevalence of post-traumatic symptoms was 0.32 (95% CI 0.26–0.37). Prevalence varied from 0.20 with PCL-C to 0.38 with IES-R. No statistical statistically significant differences regarding age, gender, or NOS score were found in meta-regressions. Sensitivity analyses found a statistically significant lower prevalence of post-traumatic symptoms in Asia (0.29; 95% CI 0.18–0.34) compared to North America (0.41; 95% CI 0.34–0.48).

### Burnout

Burnout prevalence was reported in 25 studies, including a total sample of 30,873 participants. Three scales were used to evaluate it: Mini-Z Burnout Survey (Mini-Z) [50], Copenhagen Burnout Inventory (CBI) [51], and Maslach Burnout Inventory (MBI) [52]. MBI has three measurable domains: emotional exhaustion (MBI-EE), depersonalization (MBI-DP), and reduced personal accomplishment (MBI-RPA). Scoring positively to any of these areas implies a relevant level of professional burnout. The pooled prevalence of burnout symptoms was 0.37 (95% CI 0.31–0.42). Prevalence varied from 0.22 with Mini-Z to 0.53 with CBI. In MBI, emotional exhaustion was the most deteriorated area among the sample. Sensitivity analyses and meta-regressions revealed no statistically significant differences regarding age, gender, NOS score, or continent.

### Quality assessment and meta-regressions

According to NOS Scale, the mean quality of the included studies was 5.12 ± 0.80 and ranged from three to seven. Scores for each individual article are available in Supplementary Table S4.

## Discussion

This meta-analysis has identified, for the first time on a large scale and at a global level, the prevalence of mental health symptoms in several domains in the HCWs group. HCW exposed to COVID-19 were found to have a significant prevalence rate of anxiety, depression, acute stress, insomnia, post-traumatic symptoms and burnout.

Thirty three percentage of the HCWs exposed to COVID-19 presented depressive symptomatology. This prevalence is higher than that reported in the general population during the pandemic, between 20.9% [53] and 27.8% [54]. The prevalence of anxiety in HCWs reported by this meta-analysis, 42%, is also much higher than that detected in the general population, 27.3% [55]. These results are consistent with previous findings in the literature; both Dutta et al. [56] and Saragih et al. [57] reported very similar data to those found in this study. These authors reported a total prevalence of depression of 0.32 and 0.37 and anxiety of 0.33 and 0.40, respectively. Another recent meta-analysis [58] reported a lower prevalence, 0.24 for depression and 0.26 for anxiety. However, this meta-analysis [58] included only 29 articles published in the initial months of the pandemic. All of this suggests that a progressive worsening in the mental health of HCWs may have occurred as the COVID-19 pandemic dragged on.

Insomnia was found to have a prevalence of 42%, higher than the 18–31% prevalence identified in other meta-analyses studying the general population for the same period [59–61]. This difference between the samples may be caused at least in part by the long and strenuous work shifts that characterize the duties of HCWs, which worsen insomnia and sleep quality [62,63].

As for acute stress, a prevalence of 40% was found in the sample included in our meta-analysis. While these results are similar to those previously reported in another recent meta-analysis [64], the prevalence of relevant post-traumatic stress symptoms in our sample (32%) was unexpectedly high, more than doubling the 15% prevalence previously reported [65,66]. This may be due to several reasons. Firstly, post-traumatic stress symptoms, as per definition, take time to appear, so it is reasonable to expect an increase in its prevalence as months go by. Furthermore, the previous meta-analysis included a lower number of studies, including mostly samples from Asia, limiting the generalization of its results to a global sample. General population samples also report a significantly lower prevalence of post-traumatic symptoms during the same time frame [67].

Finally, our study also analyzed the prevalence of burnout in HCWs exposed to the COVID-19 pandemic, a mental health domain little studied in previous meta-analyses. The sample included in our meta-analysis presented a 37% burnout prevalence. This is consistent with data reported by a previous study [3]. Burnout was already a relevant problem in HCWs before the COVID-19 pandemic, heavily related to a decrease in occupational well-being [68], so an increase in burnout prevalence is a growingly concerning phenomenon. During the pandemic, burnout has been especially high among young professionals due to increased workload, the loss of formational activities, and the perceived lack of proper supervision [69].

These results may have several clinical implications. First, our study confirms that HCWs are an especially vulnerable population during the COVID-19 pandemic, being more prone to mental health impairment than the general population. These findings suggest that the deterioration in the mental health of HCWs is not due to measures of general confinement, social distance and concern about the pandemic, but to the particularities of the health professions and their conditions during the pandemic. These challenges include the lack of protective equipment [70], increased workload and strenuous work shifts [71], but also ethical challenges and moral distress [72,73]. In addition to this, residents and fellows have seen their training deprioritized while also increasing their responsibility and workload [74]. Institutions should provide their professionals with proper formation, coping tools and strategies to alleviate the effects of the pandemic on their mental health. Preventive approaches should also be improved for HCWs facing these challenges, including the implementation of screening instruments to identify professionals with mental health symptomatology [3].

The meta-regression results reveal fewer symptoms in the post-traumatic domain in Asia than in other continents. Asia—and especially China, where most of the articles from this continent have been published—was initially the continent most affected by the pandemic [75], so these results may seem paradoxical. However, some studies have detected that proximity to the pandemic’s epicenter is inversely correlated with levels of distress, in a phenomenon known as the “Psychological Typhoon Eye” effect [76]. Previous research shows that deep emotional feelings, such as those related to health emergencies, decrease more quickly than less deep feelings since they activate internal psychological mechanisms designed to mitigate them [77]. Coping efficacy has been identified as a mediating factor between both events [78], which could, in turn, be stimulated by cultural factors related to collectivist cultures [79].

This study has several strengths. To the best of the authors’ knowledge, this is the largest meta-analysis published to date evaluating mental health outcomes in HCWs exposed to COVID-19. In addition, it evaluates domains of mental health less studied in previous meta-analyses, such as burnout or post-traumatic symptoms. Studies from more than 50 countries on 5 continents have been included, so its results are highly generalizable.

On the other hand, this study also has several limitations, mainly the considerable heterogeneity in the outcomes evaluated. Some authors have used different scales and cut-off points in the different domains of mental health. The exposure levels and the length of exposition duration of the HCWs included in the studied samples have not been analyzed due to the lack of data. Meta-regressions have been carried out to assess the impact of gender, professional category, and geographic origin on heterogeneity. COVID-19 pandemic has stimulated the publication of many studies in a short time, some of them of limited quality [80]. It is necessary to improve the design of the studies and standardize the methods and populations evaluated. Also, further studies should be conducted to determine in-depth the factors associated with mental health problems in HCWs during the pandemic.

In conclusion, HCWs worldwide exposed to COVID-19 were found to have a significant prevalence of concerning symptoms in a wide range of mental health domains. The effects of COVID-19 on HCWs’ mental health should not be underestimated. Further studies should be carried out to follow its evolution during the pandemic, and effective measures should be implemented to prevent and alleviate mental health deterioration in HCWs.

## Data Availability

The data that support the findings of this study are available from the corresponding author/first author, A.C./C.A., upon request.

## Supplementary material

For supplementary material accompanying this paper visit https://doi.org/10.1192/j.eurpsy.2022.1. click here to view supplementary material
