# Supplementary material for: COVID-19 pandemic effects on health worker’s mental health: Systematic review and meta-analysis
Source: Eur Psychiatry. 2022 Jan 21;65(1):e10. doi: 10.1192/j.eurpsy.2022.1 (PMC8828390; doi:10.1192/j.eurpsy.2022.1)
Supplement: Supplementary file 1 [file S0924933822000013sup001.docx]

**SUPPLEMENTARY MATERIAL**

**eTable I:** PRISMA statement and checklist……………………………………………………………………………………….................page 2-4

**eTable II:** Moose checklist……………………………………..…………………………………………………………………………….page 5-6

**eTable III:** Reasons for exclusion during full- text screening…..……………………………………………………………………..……..page 7-9

**eTable IV:** Main characteristics of the included studies …………………………………………….…………………….……....………...page 10-21

**eMethods I**: Quality assessment: Modified Newcastle-Ottawa Scale………..………………………………………………………………page 22

**eResults I:** Funnel plots …..…….………...……………………………………………………………………………. …………….….....page 23-25

**References** …..…….………...……………………………………………………………………………. ………………..………….….....page 26-46

**This supplementary material has been provided by the authors to give readers additional information about their work.**

**eTable I: Prisma statement and checklist**

| **Section/Topic** | **Item #** | **Checklist item** | **Page** |
| --- | --- | --- | --- |
| **TITLE** | | | |
| Title | 1 | Identify the report as a systematic review. | 1 |
| **ABSTRACT** |  |  |  |
| Abstract | 2 | Provide a structured summary including, as applicable: background; objectives; data sources; study eligibility criteria, participants, and interventions; study appraisal and synthesis methods; results; limitations; conclusions and implications of key findings; systematic review registration number. | 2 |
| **INTRODUCTION** | | | |
| Rationale | 3 | Describe the rationale for the review in the context of existing knowledge. | 3 |
| Objectives | 4 | Provide an explicit statement of the objective(s) or question(s) the review addresses. | 3 |
| **METHODS** | | | |
| Eligibility criteria | 5 | Specify the inclusion and exclusion criteria for the review and how studies were grouped for the syntheses. | 4-5 |
| Information sources | 6 | Specify all databases, registers, websites, organisations, reference lists and other sources searched or consulted to identify studies. Specify the date when each source was last searched or consulted. | 4 |
| Search strategy | 7 | Present the full search strategies for all databases, registers and websites, including any filters and limits used. | 4 |
| Selection process | 8 | Specify the methods used to decide whether a study met the inclusion criteria of the review, including how many reviewers screened each record and each report retrieved, whether they worked independently, and if applicable, details of automation tools used in the process. | 4-5 |
| Data collection process | 9 | Specify the methods used to collect data from reports, including how many reviewers collected data from each report, whether they worked independently, any processes for obtaining or confirming data from study investigators, and if applicable, details of automation tools used in the process. | 5 |
| Data items | 10 | List and define all outcomes for which data were sought and if any assumptions were made about any missing or unclear information. | 4-5 |
| Study risk of bias assessment | 11 | Specify the methods used to assess risk of bias in the included studies, including details of the tool(s) used, how many reviewers assessed each study and whether they worked independently, and if applicable, details of automation tools used in the process. | 4-5 |
| Effect measures | 12 | Specify for each outcome the effect measure(s) (e.g. risk ratio, mean difference) used in the synthesis or presentation of results. | 5 |
| Synthesis methods | 13 | Describe the processes used to decide which studies were eligible for each synthesis. Describe any methods required to prepare the data for presentation or synthesis, such as handling of missing summary statistics, or data conversions. Describe any methods used to tabulate or visually display results of individual studies and syntheses. Describe any methods used to synthesize results and provide a rationale for the choice(s). Describe the model(s), method(s) to identify the presence and extent of statistical heterogeneity, and software package(s) used, any methods used to explore possible causes of heterogeneity among study results (e.g. subgroup analysis, meta-regression) and any sensitivity analyses conducted to assess robustness of the synthesized results. | 5 |
| Reporting bias assessment | 14 | Describe any methods used to assess risk of bias due to missing results in a synthesis (arising from reporting biases). | 5 |
| Certainty assessment | 15 | Describe any methods used to assess certainty (or confidence) in the body of evidence for an outcome. | 5 |
| **RESULTS** | | | |
| Study selection | 16 | Describe the results of the search and selection process, from the number of records identified in the search to the number of studies included in the review, ideally using a flow diagram. Cite studies that might appear to meet the inclusion criteria, but which were excluded, and explain why they were excluded. | 6 |
| Study characteristics | 17 | Cite each included study and present its characteristics. | eTable IV |
| Risk of bias in studies | 18 | Present assessments of risk of bias for each included study. | eTable IV |
| Results of individual studies | 19 | For all outcomes, present, for each study: (a) summary statistics for each group (where appropriate) and (b) an effect estimate and its precision (e.g. confidence/credible interval), ideally using structured tables or plots. | 6-8 |
| Results of syntheses | 20 | For each synthesis, briefly summarise the characteristics and risk of bias among contributing studies. Present results of all statistical syntheses conducted. If meta-analysis was done, present for each the summary estimate and its precision (e.g. confidence/credible interval) and measures of statistical heterogeneity. If comparing groups, describe the direction of the effect. Present results of all investigations of possible causes of heterogeneity among study results and all sensitivity analyses conducted to assess the robustness of the synthesized results. | 6-8 |
| Reporting biases | 21 | Present assessments of risk of bias due to missing results (arising from reporting biases) for each synthesis assessed. | 6-8 |
| Certainty of evidence | 22 | Present assessments of certainty (or confidence) in the body of evidence for each outcome assessed. | 6-8 |
| **DISCUSSION** | | | |
| Discussion | 23 | Provide a general interpretation of the results in the context of other evidence. Discuss any limitations of the evidence included in the review, any limitations of the review processes used and the implications of the results for practice, policy, and future research. | 8-10 |
| **OTHER INFORMATION** | | | |
| Registration and protocol | 24 | Provide registration information for the review, including register name and registration number, or state that the review was not registered. Indicate where the review protocol can be accessed, or state that a protocol was not prepared. Describe and explain any amendments to information provided at registration or in the protocol. | 3 |
| Support | 25 | Describe sources of financial or non-financial support for the review, and the role of the funders or sponsors in the review. | 11 |
| Competing interests | 26 | Declare any competing interests of review authors. | 11 |
| Availability of data, code and other materials | 27 | Report which of the following are publicly available and where they can be found: template data collection forms; data extracted from included studies; data used for all analyses; analytic code; any other materials used in the review. | 3,5 |

For more information, visit: <http://www.prisma-statement.org/>

**eTable II: MOOSE Statement - Reporting Checklist for Authors, Editors, and Reviewers of Meta-analyses of Observational Studies**

| **Reporting Criteria** | **Reported (Yes/No)** | **Reported on Page** |
| --- | --- | --- |
| **Reporting of Background** |  |  |
| Problem definition | Yes | 3 |
| Hypothesis statement | Yes | 3 |
| Description of Study Outcome(s) | Yes | 4 |
| Type of exposure or intervention used | Yes | 4 |
| Type of study design used | Yes | 4 |
| Study population | Yes | 4 |
| **Reporting of Search Strategy** |  |  |
| Qualifications of searchers (eg, librarians and investigators) | Yes | 4-5 |
| Search strategy, including time period included in the synthesis and keywords | Yes | 4 |
| Effort to include all available studies, including contact with authors | Yes | 4-5 |
| Databases and registries searched | Yes | 4 |
| Search software used, name and version, including special features used (eg, explosion) | Yes | 5 |
| Use of hand searching (eg, reference lists of obtained articles) | Yes | eTable IV |
| List of citations located and those excluded, including justification | Yes | eTable III |
| Method for addressing articles published in languages other than English | Yes | 4 |
| Method of handling abstracts and unpublished studies | Yes | 4-5 |
| Description of any contact with authors | Yes | 4 |
| **Reporting of Methods** |  |  |
| Description of relevance or appropriateness of studies assembled for assessing the hypothesis to be tested | Yes | 4-5 |
| Rationale for the selection and coding of data (eg, sound clinical principles or convenience) | Yes | 4-5 |
| Documentation of how data were classified and coded (eg, multiple raters, blinding, and interrater reliability) | Yes | 5 |
| Assessment of confounding (eg, comparability of cases and controls in studies where appropriate | Yes | 5 |
| Assessment of study quality, including blinding of quality assessors; stratification or regression on possible predictors of study results YES 5 | Yes | 5 |
| Assessment of heterogeneity | Yes | 5 |
| Description of statistical methods (eg, complete description of fixed or random effects models, justification of whether  the chosen models account for predictors of study results, dose-response models, or cumulative meta-analysis) in sufficient detail to be replicated | Yes | 5 |
| Provision of appropriate tables and graphics | Yes | 11,12, Supp. |
| **Reporting of Results** |  |  |
| Table giving descriptive information for each study included | Yes | eTable IV |
| Results of sensitivity testing (eg, subgroup analysis) | Yes | 6-7 |
| Indication of statistical uncertainty of findings | Yes | 6-7 |
| **Reporting of Discussion** |  |  |
| Quantitative assessment of bias (eg, publication bias) | Yes | 10 |
| Justification for exclusion (eg, exclusion of non–English-language citations) | Yes | 9 |
| Assessment of quality of included studies | Yes | 5 |
| **Reporting of Conclusions** |  |  |
| Consideration of alternative explanations for observed results | Yes | 9-10 |
| Generalization of the conclusions (ie, appropriate for the data presented and within the domain of the literature review) | Yes | 10 |
| Guidelines for future research | Yes | 10 |
| Disclosure of funding source | Yes | 11 |

**eTable III: Reasons for exclusion during full- text screening**

| **Study** | **Reason for exclusion** | **Study** | **Reason for exclusion** |
| --- | --- | --- | --- |
| (Mira, 2020) | No desired design | (Fang, 2021) | No desired design |
| (Siyal, 2020) | No desired design | (Wang, 2020) | No desired design |
| (Cag, 2020) | No desired design | (Zang, 2021) | No desired design |
| (Menon, 2021) | No desired design | (Liu, 2020) | No desired design |
| (Corbett, 2020) | No desired design | (Elhadi, 2021) | No desired design |
| (Hacimusalar, 2020) | No desired design | (Lorente, 2021) | No desired design |
| (Li, 2020) | No desired design | (Zhongxiang, 2020) | No desired design |
| (Mohd Noor, 2021) | No desired design | (Sampaio, 2020) | No desired design |
| (Mo, 2021) | No desired design | (Tokac, 2021) | No desired design |
| (Yañez, 2020) | No desired design | (Zhang, 2021) | No desired design |
| (Reddy, 2020) | No desired design | (Yildirim, 2020) | No desired design |
| (Krasavtseva, 2020) | Language other than English | (Lee, 2020) | No desired design |
| (Cantu, 2020) | No desired design | (Chew, 2020) | No desired design |
| (Chen, 2020) | No desired design | (Vagni, 2020) | No desired population |
| (Khalafallah, 2020) | No desired design | (Asaoka, 2020) | No desired design |
| (Leskovic, 2020) | No desired design | (Karatzias, 2020) | No desired population |
| (Barello, 2020) | No desired design | (Yang, 2020) | No desired population |
| (Hu, 2021) | No desired design | (Zandifar, 2020) | No desired design |
| (Elhadi, 2020) | No desired design | (Ortega-Galán, 2020) | No desired design |
| (Miguel-Puga, 2021) | No desired design | (Trumello, 2020) | No desired design |
| (Odarushenko, 2020) | Language other than English | (Barua, 2020) | No desired design |
| (Luan, 2020) | No desired design | (Sehsah, 2021) | No desired design |
| (Ruiz-Fernández, 2020) | No desired design | (Kafle, 2021) | No desired design |
| (Abdulah, 2020) | No desired design | (Jiang, 2020) | No desired design |
| (Vagni, 2020) | No desired design | (Aksoy, 2020) | No desired design |
| (Sharif, 2020) | No desired design | (Barbore, 2020) | No desired design |
| (Arshad, 2020) | No desired design | (Sun, 2020) | No desired design |
| (Park, 2020) | No desired population | (Fu, 2021) | No desired design |
| (Wang, 2020) | No desired population | (Martínez-López, 2020) | No desired design |
| (Morgul, 2021) | No desired population | (Caillet, 2020) | No desired population |
| (Amin, 2020) | No desired design | (Ffrench-O’Carroll, 2021) | No desired design |
| (Romero, 2020) | No desired design | (Nie, 2020) | No desired design |
| (Sancak, 2020) | No desired design | (Wang, 2021) | No desired design |
| (Prazeres, 2020) | No desired design | (Jo, 2020) | No desired design |
| (Do Duy, 2020) | No desired design | (Liu, 2020) | No desired design |
| (Uvais, 2020) | No desired design | (O’Brien, 2020) | No desired population |
| (Sharma, 2020) | No desired design | (Yao, 2020) | No desired design |
| (Chen, 2020) | No desired design | (Elhadi, 2020) | No desired design |
| (Cai, 2020) | No desired design | (Lu, 2020) | No desired design |
| (Weibelzahl, 2021) | No desired population | (Jian, 2020) | Language other than English |
| (Ng, 2020) | No desired design | (Ma, 2020) | No desired design |
| (Sasaki, 2020) | No desired design | (Badahdah, 2020) | No desired design |
| (Murat, 2021) | No desired design | (Ceri, 2021) | No desired design |
| (Spiller, 2020) | No desired design | (Chang, 2020) | No desired population |
| (Simione, 2020) | No desired population | (Chieffo, 2020) | No desired design |
| (Man, 2020) | No desired design | (Zerbini, 2020) | No desired design |
| (Dincer, 2021) | No desired design | (Bassi, 2021) | No desired design |
| (Karabulut, 2021) | No desired design | (Yörük, 2021) | No desired design |
| (Usul, 2020) | No desired design | (Gázquez-Linares, 2021) | No desired design |
| (Lim, 2020) | No desired design | (Barzilay, 2020) | No desired population |
| (Cai, 2020) | No desired design | (Krok, 2021) | No desired design |
| (Mavroudis, 2021) | No desired design | (Chou, 2020) | No desired design |
| (Vujanovic, 2021) | No desired design | (Orrù, 2021) | No desired design |
| (Pasay-An, 2020) | No desired design | (Erkin, 2021) | No desired design |
| (Dinibutun, 2020) | No desired design | (Arpacioglu, 2020) | No desired population |
| (Ejeh, 2021) | No desired design | (Nowiki, 2020) | No desired design |
| (Tsehay. 2020) | No desired design | (Diomidous, 2020) | No desired design |
| (Huang, 2020) | No desired design | (Dong, 2020) | No desired design |
| (Gemine, 2021) | No desired design | (Teksin, 2020) | No desired population |
| (Vagni, 2020) | No desired design | (Talaee, 2020) | No desired design |
| (Mantri, 2020) | No desired design | (Kar, 2021) | No desired population |
| (Cunill, 2020) | No desired design | (Aljehani, 2020) | No desired design |
| (Sampaio, 2021) | No desired design | (Sorokin, 2020) | No desired population |
| (Wang, 2021) | No desired population | (Hosseinzadeh-Shanjani Z, 2020) | No desired design |
| (MacKenzie, 2021) | No desired design | (Pandey, 2021) | No desired population |
| (Tengilimoğlu, 2021) | No desired design | (Zhang, 2020) | No desired design |
| (Xie, 2020) | No desired design | (Cao, 2020) | No desired design |
| (Yi, 2021) | No desired design | (Xing, 2020) | No desired design |
| (Osman, 2020) | No desired design | (Burstyn, 2021) | No desired population |
| (Meesala, 2020) | No desired population | (Azoulay, 2020) | No desired design |
| (Secosan, 2020) | No desired design | (García, 2021) | No desired design |
| (Pinho, 2021) | No desired design | (Sunil, 2021) | No desired population |
| (Zhang, 2020) | No desired population | (Gómez-Salgado, 2021) | No desired design |
| (García-Fernández, 2020) | No desired design | (Mo, 2020) | No desired design |
| (Zhang, 2020) | No desired design | (Rodríguez-Rey, 2020) | No desired population |
| (Teng, 2020) | Language other than English | (Teng, 2020) | Language other than English |
| (Nathiya, 2021) | No desired design | (Di Tella, 2020) | No desired design |

**eTable IV: Main characteristics of the included studies**

| **Study** | **City (Country)** | **HCW involved** | **Sample size** | **Age:**  **mean** **±SD** | **Sex**  **(% female)** | **NOS Score** | **Scale (Domain)** |
| --- | --- | --- | --- | --- | --- | --- | --- |
| Liu et al., 2020 (1) | Multiple (China) | Multi-professional | 371 | N.a. | 97.7 | 6/8 | GAD-7 (Anxiety); PHQ-9 (Depression); ISI (Insomnia) |
| Wang et al., 2020 (2) | Multiple (China) | Multi-professional | 274 | 37 | 77.4 | 6/8 | GAD-7 (Anxiety); PHQ-9 (Depression); PSQI (Insomnia) |
| Santamaria et al., 2020 (3) | Multiple (Spain) | Multi-professional | 421 | N.a. | N.a. | 6/8 | DASS-21-D (Depression); DASS-21-A (Anxiety); DASS-21-S (Stress); AIS (Insomnia) |
| Si et al., 2020 (4) | Multiple (China) | Multi-professional | 863 | N.a. | 70.7 | 5/8 | DASS-21-D (Depression); DASS-21-A (Anxiety); DASS-21-S (Stress) |
| Lasalvia et al., 2020 (5) | Verona (Italy) | Multi-professional | 2076 | N.a. | 78.2 | 6/8 | SAS (Anxiety); IES-R (Postrauma); PHQ-9 (Depression) |
| Imran et al., 2020 (6) | Multiple (Pakistan) | Physicians | 10178 | 31,5±6.9 | 56.7 | 5/8 | GAD-7 (Anxiety); PHQ-9 (Depression); SASRQ (Stress) |
| Rathod et al., 2020 (7) | Multiple (United Kingdom) | Multi-professional | 3933 | N.a. | 87.5 | 6/8 | GAD-7 (Anxiety); PHQ-9 (Depression); IES-R (Postrauma) |
| Yang et al., 2021 (8) | Multiple (South Korea) | Multi-professional | 54 | N.a. | 77.8 | 4/8 | GAD-7 (Anxiety); PHQ-9 (Depression) |
| Xiao et al., 2020 (9) | Multiple (China) | Multi-professional | 958 | N.a. | 67.2 | 6/8 | HADS-D (Depression); HADS-A (Anxiety) |
| Ni et al., 2021 (10) | Wuhan (China) | Multi-professional | 54 | N.a. | 83 | 5/8 | SAS (Anxiety); SDS (Depression) |
| Feingold et al., 2021 (11) | New York City (USA) | Multi-professional | 1082 | N.a. | N.a. | 6/8 | GAD-7 (Anxiety) |
| Surrati et al., 2020 (12) | Almadinah (Saudi Arabia) | Multi-professional | 118 | N.a. | 64.5 | 5/8 | HADS-D (Depression); HADS-A (Anxiety); PSS (Stress) |
| Tan et al., 2020 (13) | Singapore (Singapore) | Multi-professional | 470 | N.a. | 68.3 | 7/8 | DASS-21-D (Depression); DASS-21-A (Anxiety); DASS-21-S (Stress) |
| Ali et al., 2020 (14) | Multiple (South Korea) | Multi-professional | 472 | 40.7 | 69.07 | 5/8 | DASS-21-D (Depression); DASS-21-A (Anxiety); DASS-21-S (Stress); IES-R (Posttrauma) |
| Que et al., 2020 (15) | Multiple (China) | Multi-professional | 913 | 33.69±7.44 | N.a. | 6/8 | GAD-7 (Anxiety); PHQ-9 (Depression); ISI (Insomnia) |
| Flateau et al., 2021 (16) | Paris (France) | Multi-professional | 353 | N.a. | 89 | 6/8 | HADS-D (Depression); HADS-A (Anxiety); IES-R (Postrauma) |
| Li et al., 2020 (17) | Wuhan (China) | Multi-professional | 4369 | N.a. | 100 | 5/8 | HADS-D (Depression); HADS-A (Anxiety); IES-R (Postrauma); GAD-7 (Anxiety); PHQ-9 (Depression) |
| Dong et al., 2021 (18) | Wuhan (China) | Multi-professional | 456 | 37.1±6.4 | N.a. | 4/8 | DASS-21-D (Depression); DASS-21-A (Anxiety); DASS-21-S (Stress) |
| Li et al., 2020-B (19) | Wuhan (China) | Multi-professional | 197 | N.a. | 75.6 | 6/8 | GAD-7 (Anxiety); PHQ-9 (Depression) |
| Ajwa et al., 2020 (20) | Riyadh (Saudi Arabia) | Multi-professional | 150 | N.a. | N.a. | 5/8 | GAD-7 (Anxiety); PHQ-9 (Depression) |
| Yildirim et al., 2020 (21) | Elazığ (Turkey) | Multi-professional | 104 | N.a. | N.a. | 4/8 | BDI (Depression); BAI (Anxiety) |
| Du et al., 2020 (22) | Wuhan (China) | Multi-professional | 134 | 36± 8.05 | 60.5 | 4/8 | PSS (Stress); BAI (Anxiety) |
| Dosil et al., 2020 (23) | Multiple (Spain) | Multi-professional | 973 | N.a. | 82.9 | 6/8 | DASS-21-D (Depression); DASS-21-A (Anxiety); DASS-21-S (Stress) |
| Holton et al., 2020 (24) | Melbourne (Australia) | Multi-professional | 391 | 42.1 ± 12.5 | 92 | 5/8 | DASS-21-D (Depression); DASS-21-A (Anxiety); DASS-21-S (Stress) |
| Monterrosa-Castro et al., 2020 (25) | Cartagena (Colombia) | Physicians | 531 | 33 ± 9.3 | 59.5 | 5/8 | GAD-7 (Anxiety) |
| Kumar et al., 2021 (26) | Karachi (Pakistan) | Multi-professional | 224 | 32 ± 3 | 24.1 | 5/8 | DASS-21-D (Depression); DASS-21-A (Anxiety); DASS-21-S (Stress) |
| Johnson et al., 2020 (27) | Multiple (Norway) | Multi-professional | 1773 | N.a. | 84.7 | 6/8 | GAD-7 (Anxiety); PHQ-9 (Depression) |
| Stojanov et al., 2020 (28) | Multiple (Serbia) | Multi-professional | 118 | 39.1 ± 7.3 | 65.6 | 4/8 | PSQI (Insomnia); SDS (Depression); GAD-7 (Anxiety) |
| Chow et al., 2021 (29) | Kuala Lumpur (Malaysia) | Multi-professional | 139 | N.a. | 60.5 | 5/8 | HADS-D (Depression); HADS-A (Anxiety) |
| Chen et al., 2021 (30) | Multiple (China) | Multi-professional | 543 | 36.41 ± 8.56 | 68.6 | 6/8 | GAD-7 (Anxiety); PHQ-9 (Depression) |
| Rodriguez-Menendez et al., 2021 (31) | Multiple (Spain) | Multi-professional | 1407 | 44.7 ± 10.9 | 73 | 6/8 | SASRQ (Anxiety) |
| HerreroSanMartin et al., 2020 (32) | Madrid (Spain) | Multi-professional | 100 | 35.3 ± 9.2 | 59 | 5/8 | PSQI (Insomnia); ISI (Insomnia) |
| Wang et al., 2020 (33) | Wuhan (China) | Multi-professional | 2001 | 33 | 64.5 | 5/8 | PSQI (Insomnia); HADS-D (Depression); HADS-A (Anxiety) |
| Wang et al., 2020-B (34) | Wuhan (China) | Multi-professional | 123 | 33.75± 8.41 | 90 | 5/8 | PSQI (Insomnia); SAS (Anxiety); SDS (Depression) |
| Alnofaiey et al., 2020 (35) | Multiple (Saudi Arabia) | Physicians | 470 | N.a. | 50.9 | 5/8 | PSQI (Insomnia) |
| Badahdah et al., 2020 (36) | Muscate (Oman) | Multi-professional | 150 | 37.62± 7.79 | 77.3 | 5/8 | SQS (Insomnia); GAD-7(Depression) |
| Zheng et al., 2020 (37) | Ningbo (China) | Multi-professional | 207 | N.a. | 84.54 | 6/8 | SQS (Insomnia) |
| Tu et al., 2020 (38) | Wuhan (China) | Nurses | 100 | 34.44 ± 5.85 | 100 | 5/8 | PSQI (Insomnia); GAD-7 (Anxiety); PHQ-9 (Depression) |
| Prasad et al., 2020 (39) | Philadelphia (USA) | Multi-Professional | 347 | N.a. | 90.8 | 5/8 | IES-R (Postrauma); GAD-7 (Depression); MINI-Z (Burnout) |
| Hennein et al., 2020 (40) | Connecticut (USA) | Multi-professional | 1092 | 40.4 ± 11.5 | 72 | 5/8 | PC-PTSD (Postrauma); GAD-7 (Anxiety); PHQ-9 (Depression) |
| Alan et al., 2020 (41) | Multiple (Turkey) | Multi-professional | 416 | 33.6±8.7 | 79.1 | 5/8 | DASS-21-D (Depression); DASS-21-A (Anxiety); DASS-21-S (Acute stress) |
| Hennein et ak., 2020 (42) | Multiple (UK) | Multi-professional | 1132 | N.a. | 71.4 | 5/8 | GAD-7 (Anxiety); PHQ-9 (Depression); PC-PTSD (Posttrauma) |
| Chen et al., 2021-B (43) | Taipei (China) | Nurses | 12596 | 33.1 | 95.6 | 4/8 | MBI-EE (Burnout); MBI-RPA (Burnout) |
| Chew et al., 2020 (44) | Multiple (Singapore, India) | Multi-professional | 906 | 29 | 64.3 | 6/8 | DASS-21-D (Depression); DASS-21-A (Anxiety); DASS-21-S (Acute stress); IES-R (Posttrauma) |
| Wang et al., 2020-C (45) | Multiple (China) | Multi-professional | 1897 | N.a. | 82.5 | 6/8 | PHQ-9 (Depression); GAD-7 (Anxiety); IES-R (Posttrauma) |
| Wang et al., 2020-D (46) | Wuhan (China) | Multi-professional | 332 | 33.2±8.8 | 78 | 6/8 | SASRQ (Acute stress); GAD-7 (Anxiety); PHQ-9 (Depression) |
| Shahrour et al., 2020 (47) | Multiple (Jordan) | Nurses | 448 | 32±8 | 73 | 3/8 | SASRQ (Acute stress) |
| Fidanci et al., 2020 (48) | Multiple (Turkey) | Multi-professional | 153 | 33.4±5.7 | 67.3 | 5/8 | PSQI (Insomnia) |
| Wu et al., 2020 (49) | Multiple (China) | Physicians | 60 | 33.8±11.9 | 75 | 4/8 | SDS (Depression); SAS (Anxiety); PSQI (Insomnia); PCL-C (Post-trauma) |
| Shen et al., 2020 (50) | Wuhan (China) | Nurses | 643 | 31.8±7.8 | 97.8 | 5/8 | GAD-7 (Anxiety); PSS (Acute Stress); AIS (Insomnia) |
| Hasan et al., 2020 (51) | Karachi (Pakistan) | Physicians | 151 | 29±7.28 | 56.3 | 5/8 | GAD-7 (Anxiety) |
| Li et al., 2020 (52) | Multiple (China) | Physicians | 5331 | N.a. | N.a. | 5/8 | GAD-7 (Anxiety); PHQ-9 (Depression) |
| Pouralizadeh et al., 2020 (53) | Multiple (Iran) | Nurses | 441 | 36.34±8.74 | 95.2 | 6/8 | GAD-7 (Anxiety); PHQ-9 (Depression) |
| Xing et al., 2020 (54) | Jinan (China) | Multi-professional | 309 | 33.3±9.5 | 97.4 | 5/8 | SAS (Anxiety); SDS (Depression) |
| Hassannia et al., 2020 (55) | Multiple (Iran) | Multi-professional | 232 | N.a. | N.a. | 6/8 | HADS-D (Depression); HADS-A (Anxiety) |
| Han et al., 2020 (56) | Multiple (China) | Nurses | 21199 | 31.89±7.084 | 98.6 | 5/8 | SAS (Anxiety); SDS (Depression) |
| Heimerer et al., 2020 (57) | Multiple (Kosovo) | Multi-professional | 592 | 39 (median) | 61.3 | 5/8 | HADS-D (Depression); HADS-A (Anxiety) |
| Setiawati et al., 2021 (58) | Surabaya (Indonesia) | Multi-professional | 227 | 39.67±9.43 | 93.3 | 3/8 | STAI-S (Anxiety) |
| Gupta et al., 2020 (59) | Multiple (India) | Multi-professional | 712 | N.a. | 54.3 | 6/8 | GAD-7 (Anxiety); SQS (Insomnia) |
| Cheng et al., 2020 (60) | Multiple (China) | Multi-professional | 534 | N.a. | 82.4 | 7/8 | SAS (Anxiety); PSQI (Insomnia) |
| Yurtseven et al., 2020 (61) | Adana (Turkey) | Nurses | 270 | 36.83±9.23 | 87.8 | 4/8 | STAI-S (Anxiety) |
| Wang et al., 2021 (62) | Nanjing (China) | Nurses | 586 | 31.07±7.54 | 96.08 | 5/8 | GAD-7 (Anxiety); PHQ-9 (Depression) |
| DiTella et all., 2021 (63) | Multiple (Italy) | Multi-professional | 73 | 44.3±10.6 | N.a. | 4/8 | STAI-S (Anxiety); BDI (Depression) |
| Awano et all., 2020 (64) | Tokyo (Japan) | Multi-professional | 461 | 43 (median) | 80.78 | 6/8 | GAD-7 (Anxiety); CES-D (Depression) |
| Simonetti et al., 2021 (64) | Multiple (Italy) | Nurses | 1005 | 40.2±10.8 | 65.97 | 6/8 | SAS (Anxiety); PSQI (Insomnia) |
| Chew et al., 2020-B (65) | Multiple (Multiple) | Multi-professional | 384 | 27.7±5.7 | 65.4 | 4/8 | DASS-21-D (Depression); DASS-21-A (Anxiety); DASS-21-S (Acute stress); IES-R (Post-trauma) |
| Arshad et., 2020 (66) | Multiple (Pakistan) | Multi-professional | 276 |  | 34.1 | 4/8 | DASS-21-D (Depression); DASS-21-A (Anxiety); DASS-21-S (Acute stress) |
| Wankowicz et al., 2020 (67) | Multiple (Poland) | Multi-professional | 206 | 40.47±4.93 | 56.31 | 6/8 | PHQ-9 (Depression); ISI (Insomnia); GAD-7 (Anxiety) |
| Lang et al., 2020 (68) | Multiple (China) | Multi-professional | 291 | 33±4 | 81 | 6/8 | GAD-7 (Anxiety); PHQ-9 (Depression) |
| Macía-Rodriguez et al., 2021 (69) | Multiple (Multiple) | Physicians | 1015 | 39.9±11.1 | 62.9 | 5/8 | MBI (Burnout) |
| Kamali et al., 2020 (70) | Shiraz (Iran) | Nurses | 261 | 28.91±6.87 | 67.2 | 5/8 | MBI (Burnout) |
| Treluyer et al., 2021 (71) | Multi (France) | Physicians | 340 | 27 | 83.8 | 5/8 | MBI (Burnout) |
| Duarte et al., 2020 (72) | Multiple (Portugal) | Multi-professional | 2008 | 38±10 | 83.6 | 5/8 | CBI (Burnout); DASS-21-D (Depression); DASS-21-A (Anxiety); DASS-21-S (Acute stress) |
| Chor et al., 2020 (73) | Singapore (Singapore) | Multi-professional | 337 | 21-30 | 67.7 | 4/8 | CBI (Burnout) |
| Tan et al., 2020 (74) | Singapore (Singapore) | Multi-professional | 2335 | 36.84±9.95 | N.a. | 7/8 | HADS-D (Depression); HADS-A; (Anxiety) |
| Dobson et al., 2020 (75) | Melbourne (Australia) | Multi-professional | 128 | N.a. | 78 | 5/8 | GAD-7 (Anxiety); IES-R (Posttrauma); PHQ-9 (Depression) |
| Jose et al., 2020 (76) | Multiple (India) | Nurses | 120 | 29±4.4 | 73.3 | 5/8 | MBI (Burnout) |
| Barello et al., 2020 (77) | Multiple (Italy) | Multi-professional | 376 | 40±11 | 73.7 | 5/8 | MBI (Burnout) |
| Sayilan et al., 2020 (78) | Multiple (Turkey) | Nurses | 267 | 28.03±5.99 | 75.3 | 5/8 | MBI (Burnout); PSQI (Insomnia) |
| Roslan et al., 2021 (79) | Multiple (Malaysia) | Multi-professional | 203 | N.a. | N.a. | 4/8 | CBI (Burnout) |
| Podder et al., 2020 (80) | Multiple (India) | Physicians | 384 | 33.7 ±9.3 | N.a. | 5 /8 | PSS (Acute stress) |
| Milgrom et al., 2020 (81) | Jerusalem (Israel) | Multi-professional | 1570 | N.a. | 71.6 | 5/8 | STAI-S (Anxiety) |
| Ahmed et al., 2021 (82) | Multiple (Egypt) | Multi-professional | 122 | N.a. | 59 | 5/8 | BDI (Depression); BAI (Anxiety) |
| Mora-Magaña et al., 2020 (83) | Multiple (Mexico) | Multi-professional | 231 | 40.35 ±10.81 | 69.3 | 5/8 | PHQ-4 (Depression) |
| Altmayer et al., 2020 (84) | Paris (France) | Multi-professional | 37 | 37 (Median) | 83 | 3/8 | HADS-D (Depression); HADS-A (Anxiety) |
| Arshad et al., 2020 (85) | Multiple (Pakistan) | Physicians | 431 | N.a. | 44.78 | 5/8 | GAD-7 (Anxiety) |
| Labrague et al., 2020 (86) | Multiple (Philipines) | Nurses | 325 | 30.94±6.76 | 74.8 | 5/8 | CAS (Anxiety) |
| Guiroy et al., 2020 (87) | Multiple (Latin America) | Physicians | 204 | 44.7 | 3.4 | 6/8 | PHQ-9 (Depression) |
| Liu et al., 2020 (88) | Multiple (China) | Multi-professional | 880 | N.a. | 68.3 | 5/8 | MBI-D (Burnout) |
| Naldi et al., 2020 (89) | Turin (Italy) | Multi-Professional | 469 | N.a. | 83.8 | 6/8 | IES-R (Posttrauma); MBI-D (Burnout) |
| Jain et al., 2020 (90) | Multiple (India) | Physicians | 512 | N.a. | 44.3 | 5/8 | GAD (Anxiety); ISI (Insomnia) |
| Saricam et al., 2020 (91) | Tekirdag (Turkey) | Nurses | 123 | 30.6±7.2 | 74 | 6/8 | STAI-S (Anxiety) |
| Mattila et al., 2020 (92) | Multiple (Finland) | Multi-Professional | 1995 | N.a. | 87 | 5/8 | GAD-7 (Anxiety) |
| Blekas et al., 2020 (93) | Multiple (Greece) | Multi-Professional | 191 | N.a. | 100 | 5/8 | PHQ-9 (Depression); PC-PTSD (Posttrauma) AIS (Insomnia) |
| Jambunathan et al, 2020 (94) | Multiple  (India) | Multi-Professional | 257 | N.a. | 42 | 4/8 | GAD-7 (Anxiety; PHQ-9 (Depression) |
| Laiyou et al., 2021 (95) | Zhejiang  (China) | Multi-Professional | 150 | N.a. | 62.7 | 5/8 | HDS (Depression); HAS (Anxiety) |
| Arafa et al., 2021 (96) | Multiple | Multi-Professional | 426 | N.a. | 49.8 | 4/8 | DASS-21-D (Depression); DASS-21-A (Anxiety); DASS-21-S (Acute stress) |
| Elbay et al., 2020 (97) | Turkey | Physicians | 442 | 36.05 ±8.69 | 56.8 | 4/8 | DASS-21-D (Depression); DASS-21-A (Anxiety); DASS-21-S (Acute stress) |
| Sunjaya et al., 2020 (98) | Multiple Indonesia) | Multi-Professional | 544 | N.a. | 77.2 | 4/8 | CES-D (Depression); SAS (Anxiety) |
| Salopek-Žiha et al., 2020 (99) | Nasice (Croatia) | Nurses | 97 | 37.85 | N.a. | 5/8 | DASS-21-D (Depression); DASS-21-A (Anxiety); DASS-21-S (Acute stress) |
| Mohd Fauzi et al., 2020 (100) | Selangor (Malaysia) | Physicians | 1050 | 33.08±6.96 | 71.5 | 4/8 | DASS-21-D (Depression); DASS-21-A (Anxiety); DASS-21-S (Acute stress) |
| Demartini et al., 2020 (101) | Lombardy (Italy) | Multi-Professional | 123 | 36±9.2 | 78.86 | 6/8 | DASS-21-D (Depression); DASS-21-A (Anxiety); DASS-21-S (Acute stress); IES-R (Posttrauma); PSQI (Insomnia); MBI-D (Burnout) |
| Erinoso et al., 2020 (102) | Lagos State (Nigeria) | Multi-Professional | 105 | 34.5±9.1 | 54.3 | 5/8 | PHQ-9 (Depression); GAD-7 (Anxiety) |
| Wasim et al., 2020 (103) | Lahore (Pakistan) | Multi-Professional | 356 | N.a. | 51.96 | 4/8 | DASS-21-D (Depression); DASS-21-A (Anxiety); DASS-21-S (Acute stress); ISI (Insomnia) |
| Marinaci et al., 2020 (104) | Salento (Italy) | Multi-Professional | 103 | 41.8±10.7 | 51.5 | 5/8 | MBI-D (Burnout) |
| Thomaier et al ., 2020 (105) | Minneapolis (USA) | Physicians | 486 | N.a. | 63.2 | 5/8 | PHQ-4 (Depression) |
| Thakre et al., 2020 (106) | India | Nurses | 232 | 38.95±4.5 | 90 | 4/8 | CAS (Anxiety) |
| Uyaroglu et al., 2020 (107) | Ankara (Turkey) | Physicians | 113 | N.a. | 46.9 | 5/8 | GAD-7 (Anxiety); BAI (Anxiety) |
| Zhan et al., 2020 (108) | Wuhan (China) | Nurses | 1794 | N.a. | 97 | 5/8 | AIS (Insomnia) |
| Alshekaili et al., 2020 (109) | Omán | Multi-Professional | 1139 | 36.3±6.5 | 80 | 3/8 | DASS-21-D (Depression); DASS-21-A (Anxiety); DASS-21-S (Acute stress) |
| Lai et al., 2020 (110) | Multiple (China) | Multi-Professional | 1257 | N.a. | 76.7 | 6/8 | PHQ-9 (Depression); GAD-7 (Anxiety); ISI (Insomnia); IES-R (Posttrauma) |
| Gu et al., 2020 (111) | Fangcang (China) | Multi-Professional | 522 | N.a. | 77.6 | 6/8 | IES-R (Posttrauma); GAD-7 (Anxiety); PHQ-9 (Depression); ISI (Insomnia); PSS (Acute stress) |
| Khoodoruth et al., 2020 (112) | Multiple (Qatar) | Physicians | 127 | N.a. | 37 | 5/8 | DASS-21-D (Depression); DASS-21-A (Anxiety); DASS-21-S (Acute stress) |
| Wang et al., 2020 (113) | Hubei (China) | Nurses | 202 | 32 | 87.6 | 5/8 | PC-PTSD (Posttrauma) |
| Jacob et al., 2020 (114) | Multiple (India) | Multi-Professional | 700 | 30.95±5.8 | 55.3 | 5/8 | DASS-21-D (Depression); DASS-21-A (Anxiety); DASS-21-S (Acute stress) |
| Li et al., 2020 (115) | Anhui (China) | Nurses | 356 | 31.3 | 86.2 | 5/8 | PSS (Acute stress); PCL-C (Posttrauma) |
| Conti et al., 2020 (116) | Italy | Multi-Professional | 933 | 41.77±12.8 | 76.5 | 5/8 | PHQ-9 (Depression); GAD-7 (Anxiety); IES-R (Posttrauma) |
| Di Monte et al., 2020 (117) | Italy | Physicians | 102 | 55.13±11.4 | 62.7 | 5/8 | MBI-D (Burnout) |
| Hu et al., 2020 (118) | Wuhan (China) | Nurses | 2014 | 30.99±6.17 | 87.1 | 5/8 | SAS (Anxiety); SDS (Depression) |
| Teshome et al., 2020 (119) | Ethiopia | Multi-Professional | 798 | 29.9±5.69 | 39.6 | 6/8 | GAD-7 (Anxiety) |
| Bahadir-Yilmaz et al., 2020 (120) | Giresun (Turkey) | Nurses | 1457 | N.a | 81 | 5/8 | SAS (Anxiety) |
| Mosolova et al., 2020 (121) | Moscow (Russia) | Multi-professional | 1090 | 35.1 **±** 11.22 | 67.9 | 5/8 | GAD-7 (Anxiety) |
| Mathur et al., 2020 (122) | Jaipur (India) | Multi-professional | 200 | 42.1 **±** 12.2 | 38 | 5/8 | DASS-21-S (Acute stress); DASS-21-D (Depression); DASS-21-A (Anxiety) |
| Xu et al., 2020 (123) | Chongqing (China) | Multi-professional | 11507 | 33.37 ± 8.22 | 78.09 | 5/8 | PHQ-9 (Depression); GAD-7 (Anxiety) |
| Zhang et al., 2020 (124) | Multiple (China) | Multi-professional | 1563 | N.a | 82.72 | 5/8 | ISI (insomnia); PHQ-9 (Depression); GAD-7 (Anxiety) |
| Yaoyao et al., 2020 (125) | Multiple (China) | Multi-professional | 127 | 32 ± n.a | 62.2 | 6/8 | PHQ-9 (Depression); SAS (Anxiety) |
| Gupta et al., 2020 (126) | Bangalore (India) | Multi-professional | 1124 | N.a | 36.1 | 6/8 | HADS-A (Anxiety) |
| Azoulay et al., 2020 (127) | Multiple (France) | Multi-professional | 1058 | 33 | 71 | 6/8 | HADS-A (Anxiety); HADS-D (Depression) |
| Luceño-Moreno et al., 2020 (128) | Multiple (Spain) | Multi-professional | 1422 | 43.88 ± 10.82 | 86.4 | 6/8 | IES-R (Postrauma); HADS-A (Anxiety); HADS-D (Depression); MBI (Burnout) |
| Young et al., 2020 (129) | Multiple (USA) | Physicians | 1685 | N.a | 76 | 5/8 | GAD-7 (Anxiety); PHQ-9 (Depression); PC-PTSD (Postrauma) |
| Korkmaz et al., 2020 (130) | Multiple (Turkey) | Multi-professional | 140 | N.a | 44 | 7/8 | BAI (Anxiety) |
| Mosheva et al., 2021 (131) | Multiple (Israel) | Multi-professional | 639 | 43.1 ± 11.3 | 69.3 | 5/8 | PHQ-9 (Depression); PC-PTSD (Postrauma) |
| Rapisarda et al., 2020 (132) | Lombard (Italy) | Multi-professional | 241 | 44.2 ± 12.3 | 76.8 | 5/8 | PHQ-9 (Depression); GAD-7 (Anxiety); MBI (Burnout) |
| Crowe et al., 2020 (133) | Surrey (Canada) | Nurses | 43952 | 37.1 ± 89.9 | N.a | 6/8 | IES-R (Postrauma); DASS-21-D (Depression) |
| Secdegul et al., 2020 (134) | Ankara (Turkey) | Multi-professional | 308 | N.a | N.a | 6/8 | DASS-21-A (Anxiety); DASS-21-D (Depression); DASS-21-S (Acute Stress) |
| Çaliskan et al., 2020 (135) | Samsun (Turkey) | Physicians | 290 | N.a | N.a | 5/8 | HADS-A (Anxiety); HADS-D (Depression) |
| Qi et al., 2020 (136) | Hubei (China) | Physicians | 801 | 33.1 ± 8.4 | 79.9 | 3/8 | AIS (Insomnia); PSQI (Insomnia); |
| Jahrami et al., 2020 (137) | Bahrain (Bahrain) | Multi-professional | 257 | N.a | N.a | 5/8 | PSS (Acute Stress); PSQI (Insomnia) |
| Karasu et al., 2020 (138) | Multiple (Turkey) | Multi-professional | 710 | 33.9 ± 8.56 | 60.7 | 5/8 | STAI-S (Anxiety) |
| Geng et al., 2021 (139) | Multiple (China) | Multi-professional | 317 | N.a | 69.7 | 6/8 | PCL-C (Postrauma) |
| Lin et al., 2020 (140) | Hubei (China) | Multi-professional | 885 | N.a | N.a | 4/8 | PHQ-9 (Depression); GAD-7 (Anxiety); ISI (Insomnia) |
| Khamis et al., 2020 (141) | Multiple (Oman) | Multi-professional | 402 | 36.4 ± 6.7 | 100 | 4/8 | PSS (Acute Stress); SQS (Insomnia); GAD-7 (Anxiety) |
| Cai et al., 2020 (142) | Multiple (China) | Multi-professional | 1173 | 30.6 ± 8.8 | 69.8 | 6/8 | PHQ-9 (Depression); BAI (Anxiety); ISI (Insomnia) |
| Badahdah et al., 2020 (143) | Multiple (Oman) | Multi-professional | 509 | 37.67 ± 7.68 | 80.3 | 5/8 | GAD-7 (Anxiety); PSS (Acute Stress) |
| Ning et al., 2020 (144) | Multiple (China) | Multi-professional | 612 | N.a | 72.9 | 4/8 | SAS (Anxiety); SDS (Depression) |
| Ide et al., 2021 (145) | Yokohama (Japan) | Multi-professional | 2697 | N.a | 75 | 6/8 | IES-R (Acute Stress) |
| Li et al., 2020 (146) | Guangdong (China) | Multi-professional | 908 | 33.8 ± 6.93 | 75.55 | 6/8 | SAS (Anxiety); SDS (Depression) |
| Saracoglu et al., 2020 (147) | Multiple (Turkey) | Multi-professional | 208 | 29 ± 7.74 | 27.9 | 5/8 | PSQI (Insomnia); PHQ-9 (Depression) |
| Teo et al., 2021 (148) | Singapur (Singapur) | Multi-professional | 122 | 34 | 73.8 | 6/8 | GAD-7 (Anxiety); SDS (Depression) |
| Castelli et al., 2021 (149) | Piedmont (Italy) | Multi-professional | 246 | 51.1 ± 13.1 | 56 | 5/8 | STAI (Anxiety); BDI (Depression); PCL-C (Postrauma) |
| Chen et al., 2020 (150) | Wuhan (China) | Multi-professional | 422 | N.a | N.a | 6/8 | SDS (Depression); IES-R (Posttrauma); PSS (Acute Stress) |
| Mekonen et al., 2020 (151) | Multiple (Ethiopia) | Nurses | 293 | 29.6 ± 5.1 | 45.4 | 7/8 | DASS-21-D (Depression); DASS-21-A (Anxiety); DASS-21-S (Stress). |
| Wang et al., 2020 (152) | Guangdong (China) | Multi-professional | 1049 | N.a | 85.8 | 5/8 | HADS-A (Anxiety); HADS-D (Depression); ISI (Insomnia) |
| Giusti et al., 2020 (153) | Multiple (Italy) | Multi-professional | 330 | 44.6 ± 13.5 | 62.6 | 7/8 | DASS-21-D (Depression); DASS-21-A (Anxiety); DASS-21-S (Acute Stress); STAI-S (Anxiety); IES-R (Posttrauma); MBI (Burnout) |
| Al Mahyjari et al., 2020 (154) | Multiple (Oman) | Multi-professional | 91 | N.a | 74.1 | 5/8 | GAD-7 (Anxiety); PSS (Acute Stress) |
| Xiong et al., 2020 (155) | Xiamen (China) | Nurses | 361 | N.a | 97.3 | 6/8 | GAD-7 (Anxiety); PHQ-9 (Depression) |
| Xiaoming et al., 2020 (156) | Chongqing (China) | Multi-professional | 8817 | 33.25±8.26 | 78 | 6/8 | GAD-7 (Anxiety); PHQ-9 (Depression) |
| Li et al., 2020 (157) | Multiple (China) | Multi-professional | 606 | 35.77± 8.13 | 81.2 | 5/8 | GAD-7 (Anxiety); ISI (Insomnia) |
| Xia et al., 2021 (158) | Tangshan (China) | Multi-professional | 126 | 34.98± 8.03 | 69.05 | 5/8 | PHQ-9 (Depression) |
| Teshome et al, 2020 (159) | Multiple (Ethiopia) | Multi-professional | 798 | 29.29±5.69 | 39.6 | 6/8 | GAD-7 (Anxiety) |
| Young et al, 2020 (160) | Multiple (USA) | Multi-professional | 1685 | N.a. | 76 | 5/8 | PHQ-9 (Depression); GAD-7 (Anxiety); PC-PTSD (Posttrauma) |
| Amra et al, 2021 (161) | Isfashan (Iran) | Multi-professional | 372 | 345 | 7.1 | 6/8 | PHQ-9 (Depression); GAD-7 (Anxiety); ISI (Insomnia) |
| Halayem et al, 2020 (162) | Multiple (Tunisia) | Physicians | 191 | 33±7.9 | 80.9 | 4/8 | PSS (Acute stress) |
| Hong et al, 2020 (163) | Multiple (China) | Nurses | 4692 | N.a. | 96.9 | 5/8 | PHQ-9 (Depression); GAD-7 (Anxiety) |
| Cui et al, 2021 (164) | Jiangsu (China) | Multi-professional | 453 | 33.15±8.38 | 96.47 | 5/8 | SAS (Anxiety); PSS (Acute stress) |
| Sandesh et al, 2020 (165) | Karachi (Pakistan) | Multi-professional | 204 | N.a. | N.a. | 3/8 | DASS-21-D (Depression); DASS-21-A (Anxiety); DASS-21-S (Acute Stress) |
| Chatzittofis et al, 2021 (166) | Multiple (Cyprus) | Multi-professional | 424 | 38.78±11.40 | 58.5 | 6/8 | PHQ-9 (Depression); IES-R (Posttrauma); PSS (Acute stress) |
| Kang et al, 2020 (167) | Wuhan (China) | Multi-professional | 944 | N.a. | 85.5 | 5/8 | PHQ-9 (Depression); IES-R (Posttrauma); GAD-7 (Anxiety); ISI (Insomnia) |
| Tran et al, 2020 (168) | Multiple (Vietnam) | Multi-professional | 7124 | 34.4±8.8 | 66.2 | 6/8 | PHQ-9 (Depression); GAD-7 (Anxiety) |
| Zhang et al, 2020 (169) | Wuhan (China) | Multi-professional | 966 | N.a. | 76.4 | 6/8 | PSS (Acute stress); GAD-7 (Anxiety); PHQ-9 (Depression) |
| Sagherian et al, 2020 (170) | Wisconsin (USA) | Multi-professional | 587 | N.a. | 94.06 | 5/8 | ISI (Insomnia); PHQ-9 (Depression) |
| Tselebis et al, 2020 (171) | Athens (Greece) | Nurses | 150 | 42.29±1.73 | 80.01 | 5/8 | AIS (Insomnia); PSS (Acute stress) |
| Shen et al, 2020 (172) | Wuhan (China) | Nurses | 643 | 31.8±7.8 | 97.8 | 4/8 | AIS (Insomnia); PSS (Acute stress); GAD-7 (Anxiety) |
| An et al, 2021 (173) | Diyarbakir (Turkey) | Multi-professional | 198 | N.a. | N.a. | 3/8 | HADS-D (Depression); HADS-A (Anxiety) |
| Zhao et al, 2020 (174) | Multiple (China) | Multi-professional | 215 | N.a. | 76.29 | 6/8 | PSQI (Insomnia) |
| Jindal et al, 2020 (175) | Punjab (India) | Multi-professional | 296 | N.a. | N.a. | 6/8 | GAD-7 (Anxiety) |
| Lasalvia et al, 2021 (176) | Verona (Italia) | Multi-professional | 1961 | N.a. | 74.8 | 5/8 | MBI (Burnout) |
| Roberts et al, 2020 (177) | Multiple (United Kingdom) | Multi-professional | 255 | 45.1±9.77 | 88.6 | 5/8 | GAD-7 (Anxiety); PHQ-9 (Depression) |
| Leng et al, 2020 (178) | Wuhan (China) | Nurses | 90 | N.a. | 72.2 | 4/8 | PCL-C (Posttrauma); PSS (Acute stress) |
| Civantos et al, 2020 (179) | Multiple (Brazil) | Physicians | 163 | N.a. | 25.8 | 5/8 | GAD-7 (Anxiety); IES-R (Posttrauma); Mini-Z (Burnout) |
| AlAteeq et al, 2020 (180) | Multiple (Saudi Arabia) | Multi-professional | 502 | 35±N.a. | 31.9 | 5/8 | PHQ-9 (Depression); GAD-7 (Anxiety) |
| Hummel et al, 2020 (181) | Multiple (Multiple) | Multi-professional | 609 | 41±N.a. | 75.2 | 5/8 | DASS-21-D (Depression) |
| Manh et al, 2020 (182) | Hanoi (Vietnam) | Multi-professional | 173 | 31±N.a. | 68.2 | 5/8 | DASS-21-D (Depression); DASS-21-A (Anxiety); DASS-21-S (Acute Stress) |
| Suryavansh et al, 2020 (183) | Maharashtra (India) | Multi-professional | 197 | N.a. | 51 | 5/8 | GAD-7 (Anxiety); PHQ-9 (Depression) |
| Gorini et al, 2020 (184) | Lombardy (Italy) | Multi-professional | 650 | 44±N.a. | 71 | 5/8 | IES-R (Posttrauma) |
| Tian et al, 2020 (185) | Beijing (China) | Multi-professional | 845 | 35.5±6.7 | 84.5 | 4/8 | PSS (Acute stress); GAD-7 (Anxiety); PHQ-9 (Depression); ISI (Insomnia) |
| Qian et al, 2019 (186) | Multiple (China) | Multi-professional | 403 | 32±N.a. | 77.4 | 5/8 | PHQ-9 (Depression); GAD-7 (Anxiety); PSQI; (Insomnia) |
| Alonso et al, 2020 (187) | Multiple (Spain) | Multi-professional | 9138 | 39±N.a. | N.a. | 5/8 | PHQ-9 (Depression); GAD-7 (Anxiety) |
| Khanal et al, 2020 (188) | Multiple (Nepal) | Multi-professional | 475 | 28.20±5.8 | 56.2 | 5/8 | ISI (Insomnia) |
| Liang et al, 2020 (189) | Multiple (China) | Multi-professional | 899 | 35.2±N.a. | 18.69 | 4/8 | GAD-7 (Anxiety); PHQ-9 (Depression); ISI (Insomnia) |
| Elkholy et al, 2020 (190) | Multiple (Egypt) | Multi-professional | 502 | N.a. | 50 | 6/8 | PHQ-9 (Depression); GAD-7 (Anxiety); ISI (Insomnia) |
| Yitayih et al, 2020 (191) | Multiple (Ethiopia) | Multi-professional | 249 | 27.4±4.1 | 52.6 | 5/8 | IES-R (Posttrauma); ISI (Insomnia) |
| Antonijevic et al, 2020 (192) | Multiple (Serbia) | Physicians | 1678 | 40.38±10.32 | 78.  6 | 5/8 | GAD-7 (Anxiety); BDI (Depression) |
| Blewussi et al, 2020 (193) | Multiple (Togo) | Multi-professional | 62 | 35.5±8.75 | 56.5 | 4/8 | GAD-7 (Anxiety); PHQ-9 (Depression) |
| Bassani et al, 2020 (194) | Paraná (Brazil) | Nurses | 88 | 21±N.a. | 89.8 | 6/8 | HADS-A (Anxiety); HADS-D (Depression) |
| Jokic-Begic et al, 2020 (195) | Zagreb (Croatia) | Multi-professional | 725 | 48.3±11.26 | 71.9 | 4/8 | CAS (Anxiety) |
| Greenberg et al, 2020 (196) | Multiple (United Kingdom) | Multi-professional | 709 | N.a. | N.a. | 5/8 | GAD-7 (Anxiety); PHQ-9 (Depression); PCL-C (Posttrauma) |
| Rossi et al., 2020 (197) | Multiple  (Italy) | multi-profesional | 1379 | 39±16 | 77.2 | 6/8 | PSS (Acute stress); GAD-7 (Anxiety); PHQ-9 (Depressión); ISI-7 (Insomnia) |
| Rossi et al., 2020 (198) | Multiple (Italy) | Physicians | 1411 | 40.64±10.28 | 79.73 | 6/8 | PHQ-9 (Depression); GAD-7 (Anxiety); ISI-7 (Insomnia) |
| Al Ammari et al., 2020 (199) | Multiple (Saudi Arabia) | Multi-professional | 722 | 43.2±9.5 | 64.17 | 5/8 | PHQ-9 (Depression); GAD-7 (Anxiety); ISI-7 (Insomnia) |
| Tiete et al., 2020 (200) | Multiple (Belgium) | Multi-professional | 647 | 45.3±15.2 | 78.4 | 6/8 | GAD-7 (Anxiety); ISI-7 (Insomnia); PHQ-9 (Depression); DASS-21-S (Acute stress) |
| Robles et al., 2020 (201) | Multiple (Mexico) | Multi-professional | 5938 | 39.6±11.9 | 74.4 | 4/8 | PHQ-2 (Depression) |
| Szepietowski et al., 2020 (202) | Wrocklaw (Poland) | Multi-professional | 62 | 44.4±11.9 | 78.3 | 4/8 | GAD-7 (Anxiety); PHQ-9 (Depression); HADS-D (Depresson); HADS-A (Anxiety); |
| Youssef et al., 2020 (203) | Multiple (Egypt) | Multi-professional | 540 | 37.3±9.2 | 45.6 | 6/8 | ISI-7 (Insomnia); DASS-21-S (Acute stress); DASS-21-a (Anxiety); DASS-21-D (Depression) |
| Song et al., 2020 (204) | Multiple (China) | Multi-professional | 14825 | 34±8.2 | 64.3 | 5/8 | CES-D (Depression); PC-PTSD (Post-trauma) |
| Liu et al., 2020 (205) | Multiple (China) | Multi-professional | 1173 | N.a. | 85.52 | 6/8 | DASS-21-D (Depression); DASS-21-A (Anxiety); DASS-21-S (Acute stress) |
| Naser et al., 2020 (206) | Multiple (Jordan) | Multi-professional | 1163 | N.a. | 56.1 | 5/8 | PHQ-9 (Depression); GAD-7 (Anxiety) |
| Wanigasooriya et al., 2020 (207) | Multiple (United Kingdom) | Multi-professional | 2638 | 42 | 79.5 | 5/8 | PHQ-4 (Depression); PHQ-4 (Anxiety); IES-R (Post-trauma) |
| Huang et al., 2020 (208) | Wuhan (China) | Nurses | 966 | 31.92±5.89 | 91.3 | 5/8 | PSQI (Insomnia) |
| Havaei et al., 2020 (209) | Multiple (Canada) | Nurses | 3676 | N.a. | N.a. | 5/8 | GAD-7 (Anxiety); PHQ-9 (Depression); |
| Vafaei et al., 2020 (210) | Multiple (Iran) | Physicians | 194 | N.a. | 32.4 | 5/8 | PHQ-9 (Depression) |
| Zhang et al., 2020 (211) | Wenzhou (China) | Multi-professional | 150 | 33.63±6.72 | 86 | 5/8 | PHQ-9 (Depression); GAD-7 (Anxiety); ISI-7 (Insomnia) |
| Sun et al., 2020 (212) | Multiple (China) | Multi-professional | 536 | 36.2±9.63 | 69 | 5/8 | PHQ-9 (Depression); GAD-7 (Anxiety) |
| Wright et al., 2020 (213) | Multiple (USA) | Multi-professional | 98 | 42.93±11.02 | N.a. | 5/8 | GAD-7 (Anxiety); |
| Lam et al., 2020 (214) | Multiple (China) | Multi-professional | 510 | N.a. | 74.7 | 5/8 | PHQ-9 (Depression) |
| Arnetz et al., 2020 (215) | Michigan (USA) | Nurses | 695 | N.a. | 93.6 | 5/8 | PHQ-9 (Depression); GAD-7 (Anxiety); PC-PTSD (Post-trauma) |
| Haravuori et al., 2020 (216) | Helsinki (Finland) | Multi-professional | 4804 | 44.2±11.4 | 87.5 | 6/8 | ISI (Insomnia); PC-PTSD (Post-trauma); PHQ-2 (Depression) |
| Marco et al., 2020 (217) | Multiple (USA) | Physicians | 1300 | 47.4±0.33 | 23.4 | 4/8 | PC-PTSD (Post-trauma) |
| Zhang et al., 2020 (218) | Wuhan (China) | Multi-professional | 642 | N.a. | 85.05 | 7/8 | PC-PTSD (Post-trauma); HADS-D (Depression); HADS-A (Anxiety); ISI (Insomnia) |
| Yin et al., 2020 (219) | Multiple (China) | Multi-professional | 371 | 35.3±9.48 | 61.5 | 4/8 | PC-PTSD (Post-trauma) |
| Pang et al., 2020 (220) | Multiple (China) | Nurses | 282 | N.a. | 88.6 | 5/8 | GAD-7 (Anxiety); PHQ-9 (Depression) |
| Kim et al., 2020 (221) | Multiple (USA) | Nurses | 320 | 33 | 94.4 | 6/8 | GAD-7 (Anxiety); PSS (Acute stress); PHQ-9 (Depression) |
| Zheng et al., 2021 (222) | Hubei (China) | Nurses | 617 | 40 | 99.5 | 4/8 | DASS-21-d (Depression); DASS-21-A (Anxiety); DASS-21-S (Acute stress) |
| Zhou et al., 2020 (223) | Multiple (China) | Multi-professional | 1931 | 35.08±8.04 | 83.6 | 5/8 | PSQI (Insomnia); |
| Zhu et al., 2020 (224) | Gansu province (China) | Multi-professional | 165 | 34.16±8.06 | 83 | 5/8 | SAS (Anxiety); SDS (Depression) |
| Zhan et al., 2020 (225) | Wuhan (China) | Nurses | 2667 | 30 | 96.96 | 6/8 | GAD-7 (Anxiety); PHQ-9 (Depression) |
| Labrague et al., 2020 (226) | Multiple (Philippines) | Nurses | 736 | 30.9±6.77 | 71.3 | 4/8 | CAS (Anxiety) |
| Vallee et al., 2020 (227) | Multiple (France) | Physicians | 1001 | N.a. | 51.6 | 6/8 | GAD-7 (Anxiety); PHQ-9 (Depression); ISI (Insomnia) |
| Yu et al., 2020 (228) | Multiple (China) | Multi-professional | 290 | 31 | 64.1 | 5/8 | GAD-7 (Anxiety) |
| Skoda et al., 2020 (229) | Multiple (Germany) | Multi-professional | 2009 | N.a. | 75.48 | 5/8 | GAD-7 (Anxiety) |
| Keubo et al., 2021 (230) | Multiple (Cameroon) | Multi-professional | 292 | N.a. | 54.5 | 5/8 | HADS-D (Depression); HADS-A (Anxiety) |
| Park et al., 2020 (231) | Multiple (South Korea) | Physicians | 151 | 41 | 58.3 | 4/8 | DASS-21-D (Depression); DASS-21-A (Anxiety); DASS-21-S (Acute stress) |
| Lee et al., 2020 (232) | Multiple (Singapore) | Multi-professional | 270 | 30 | 70.7 | 5/8 | HADS-D (Depression); HADS-A (Anxiety) |
| Bizri et al., 2021 (233) | Beirut (Lebanon) | Multi-professional | 150 | N.a. | 56 | 5/8 | PSS (Acute stress); IES-R (Post-trauma) |
| Juan et al., 2020 (234) | Chongqing (China) | Multi-professional | 456 | 30.67±7.48 | 70.6 | 6/8 | IES-R (Post-trauma); GAD-7 (Anxiety); PHQ-9 (Depression) |
| Gupta et al., 2020 (235) | Multiple (India) | Physicians | 749 | N.a. | 25.8 | 5/8 | HADS-A (Anxiety); HADS-D (Depression) |
| Tan et al., 2021 (236) | Multiple (Multiple) | Multi-professional | 3391 | N.a. | 30 | 5/8 | DASS-21-A (Anxiety); DASS-21-D (Depression); DASS-21-S (Acute stress); IES-R (Post-trauma) |

**eMethods I: Quality assessment: Modified Newcastle-Ottawa Scale (NOS).**

Quality of the included studies was assessed using a modified version of the Newcastle-Ottawa Scale (NOS) due to the heterogeneity expected in the included studies. The following assessment scores were used:

| **Criteria** | **Maximum Score** |
| --- | --- |
| Representativeness of exposed cohort | ★ |
| Method used to ascertain exposure is robust? | ★ |
| Is there an adjustment for confounding factors? | ★ ★ |
| Assessment of outcome was blind to exposure status or used record linkage, were robust tools used? | ★ ★ |
| Exposure period was sufficiently long for outcomes to occur? | ★ |
| Loss to follow-up rate is reported a low (<30%)? | ★ |

**eResults I: Funnel plots.**

eResults I-B: Depression Funnel Plot

eResults I-A: Burnout Funnel Plot

eResults I-D: Posttrauma Funnel Plot

eResults I-C: Acute Stress Funnel Plot

eResults I-F: Anxiety Funnel Plot

eResults I-E: Insomnia Funnel Plot

**References**

(1) Liu T, Zheng Z, Sha X, Liu H, Zheng W, Su H, et al. Psychological impact in non-infectious disease specialists who had direct contact with patients with COVID-19. BJPsych Open 2020 December 07;7(1):e8.

(2) Wang LQ, Zhang M, Liu GM, Nan SY, Li T, Xu L, et al. Psychological impact of coronavirus disease (2019) (COVID-19) epidemic on medical staff in different posts in China: A multicenter study. J Psychiatr Res 2020 October 01;129:198-205.

(3) Dosil Santamaria M, Ozamiz-Etxebarria N, Redondo Rodriguez I, Jaureguizar Alboniga-Mayor J, Picaza Gorrotxategi M. Psychological impact of COVID-19 on a sample of Spanish health professionals. Rev Psiquiatr Salud Ment (Engl Ed) 2021 June 01;14(2):106-112.

(4) Si MY, Su XY, Jiang Y, Wang WJ, Gu XF, Ma L, et al. Psychological impact of COVID-19 on medical care workers in China. Infect Dis Poverty 2020 August 12;9(1):113-0.

(5) Lasalvia A, Bonetto C, Porru S, Carta A, Tardivo S, Bovo C, et al. Psychological impact of COVID-19 pandemic on healthcare workers in a highly burdened area of north-east Italy. Epidemiol Psychiatr Sci 2020 December 17;30:e1.

(6) Imran N, Masood HMU, Ayub M, Gondal KM. Psychological impact of COVID-19 pandemic on postgraduate trainees: a cross-sectional survey. Postgrad Med J 2020 August 25.

(7) Rathod S, Pallikadavath S, Young AH, Graves L, Rahman MM, Brooks A, et al. Psychological impact of COVID-19 pandemic: Protocol and results of first three weeks from an international cross-section survey - focus on health professionals. J Affect Disord Rep 2020 December 01;1:100005.

(8) Yang S, Kwak SG, Chang MC. Psychological impact of COVID-19 on hospital workers in nursing care hospitals. Nurs Open 2020 September 20.

(9) Xiao X, Zhu X, Fu S, Hu Y, Li X, Xiao J. Psychological impact of healthcare workers in China during COVID-19 pneumonia epidemic: A multi-center cross-sectional survey investigation. J Affect Disord 2020 September 01;274:405-410.

(10) Ni J, Wang F, Liu Y, Wu M, Jiang Y, Zhou Y, et al. Correction: Psychological Impact of the COVID-19 Pandemic on Chinese Health Care Workers: Cross-Sectional Survey Study. JMIR Ment Health 2021 February 02;8(2):e27596.

(11) Feingold JH, Peccoralo L, Chan CC, Kaplan CA, Kaye-Kauderer H, Charney D, et al. Psychological Impact of the COVID-19 Pandemic on Frontline Health Care Workers During the Pandemic Surge in New York City. Chronic Stress (Thousand Oaks) 2021 February 01;5:2470547020977891-Dec.

(12) Amal M. Qasem Surrati, SBFM,a,∗ Farah M. Asad Mansuri, FCPS,a and Abeer A. Ayadh Alihabi. Psychological impact of the COVID-19 pandemic on health care workers. J Taibah Univ Med Sci 2020 Nov 5,:536–543.

(13) Tan YQ, Chan MT, Chiong E. Psychological health among surgical providers during the COVID-19 pandemic: a call to action. Br J Surg 2020 October 01;107(11):e459-e460.

(14) Ali S, Maguire S, Marks E, Doyle M, Sheehy C. Psychological impact of the COVID-19 pandemic on healthcare workers at acute hospital settings in the South-East of Ireland: an observational cohort multicentre study. BMJ Open 2020 December 18;10(12):e042930-042930.

(15) Que J, Shi L, Deng J, Liu J, Zhang L, Wu S, et al. Psychological impact of the COVID-19 pandemic on healthcare workers: a cross-sectional study in China. Gen Psychiatr 2020 June 14;33(3):e100259-100259. eCollection 2020.

(16) Flateau C, Noel C, Bonnafoux A, Fuentes E, de Pontfarcy A, Diamantis S. Psychological impact of the SARS-CoV-2 outbreak on the staff of a French hospital. Infect Dis Now 2021 March 01;51(2):187-193.

(17) Li G, Miao J, Wang H, Xu S, Sun W, Fan Y, et al. Psychological impact on women health workers involved in COVID-19 outbreak in Wuhan: a cross-sectional study. J Neurol Neurosurg Psychiatry 2020 August 01;91(8):895-897.

(18) Dong LN, Zhao YL, Liu Y, Wu YZ, Wang JP. Psychological reactions of healthcare workers deployed to Wuhan from Shanxi province and how they cope during coronavirus disease 2019 (COVID-19) outbreak. Chin Med J (Engl) 2021 January 05;134(5):587-589.

(19) Li XY, Wang J, Zhang RX, Chen L, He CK, Wang CY, et al. Psychological Status Among Anesthesiologists and Operating Room Nurses During the Outbreak Period of COVID-19 in Wuhan, China. Front Psychiatry 2020 December 03;11:574143.

(20) Ajwa N, Al Rafee A, Al Rafie H, Alrafee N, Alduhaimi N, Zainaldeen F, et al. Psychological status assessment of medical and dental staff during the covid-19 outbreak in Saudi Arabia. Medical Science ;24(106):4790-4797.

(21) Yildirim TT, Atas O, Asafov A, Yildirim K, Balibey H. Psychological Status of Healthcare Workers during the Covid-19 Pandemic. J Coll Physicians Surg Pak 2020 June 01;30(6):26-31.

(22) Du J, Dong L, Wang T, Yuan C, Fu R, Zhang L, et al. Psychological symptoms among frontline healthcare workers during COVID-19 outbreak in Wuhan. Gen Hosp Psychiatry 2020 December 01;67:144-145.

(23) Dosil M, Ozamiz-Etxebarria N, Redondo I, Picaza M, Jaureguizar J. Psychological Symptoms in Health Professionals in Spain After the First Wave of the COVID-19 Pandemic. Front Psychol 2020 December 18;11:606121.

(24) Holton S, Wynter K, Trueman M, Bruce S, Sweeney S, Crowe S, et al. Psychological well-being of Australian hospital clinical staff during the COVID-19 pandemic. Aust Health Rev 2021 June 01;45(3):297-305.

(25) Monterrosa-Castro A, Redondo-Mendoza V, Mercado-Lara M. Psychosocial factors associated with symptoms of generalized anxiety disorder in general practitioners during the COVID-19 pandemic. J Investig Med 2020 October 01;68(7):1228-1234.

(26) Kumar D, Saghir T, Ali G, Yasin U, Furnaz S, Karim M, et al. Psychosocial Impact of COVID-19 on Healthcare Workers at a Tertiary Care Cardiac Center of Karachi Pakistan. J Occup Environ Med 2021 February 01;63(2):e59-e62.

(27) Johnson SU, Ebrahimi OV, Hoffart A. PTSD symptoms among health workers and public service providers during the COVID-19 outbreak. PLoS One 2020 October 21;15(10):e0241032.

(28) Stojanov J, Malobabic M, Stanojevic G, Stevic M, Milosevic V, Stojanov A. Quality of sleep and health-related quality of life among health care professionals treating patients with coronavirus disease-19. Int J Soc Psychiatry 2021 March 01;67(2):175-181.

(29) Chow SK, Francis B, Ng YH, Naim N, Beh HC, Ariffin MAA, et al. Religious Coping, Depression and Anxiety among Healthcare Workers during the COVID-19 Pandemic: A Malaysian Perspective. Healthcare (Basel) 2021 January 15;9(1):10.3390/healthcare9010079.

(30) Chen J, Liu X, Wang D, Jin Y, He M, Ma Y, et al. Risk factors for depression and anxiety in healthcare workers deployed during the COVID-19 outbreak in China. Soc Psychiatry Psychiatr Epidemiol 2021 January 01;56(1):47-55.

(31) Gonzalo RM, Ana RG, Patricia CA, Laura AL, Nathalia GT, Luis C, et al. Short-term emotional impact of COVID-19 pandemic on Spaniard health workers. J Affect Disord 2021 January 01;278:390-394.

(32) Herrero San Martin A, Parra Serrano J, Diaz Cambriles T, Arias Arias EM, Munoz Mendez J, Del Yerro Alvarez, M J, et al. Sleep characteristics in health workers exposed to the COVID-19 pandemic. Sleep Med 2020 November 01;75:388-394.

(33) Wang W, Song W, Xia Z, He Y, Tang L, Hou J, et al. Sleep Disturbance and Psychological Profiles of Medical Staff and Non-Medical Staff During the Early Outbreak of COVID-19 in Hubei Province, China. Front Psychiatry 2020 July 22;11:733.

(34) Wang S, Xie L, Xu Y, Yu S, Yao B, Xiang D. Sleep disturbances among medical workers during the outbreak of COVID-2019. Occup Med (Lond) 2020 July 17;70(5):364-369.

(35) Alnofaiey YH, Alshehri HA, Alosaimi MM, Alswat SH, Alswat RH, Alhulayfi RM, et al. Sleep disturbances among physicians during COVID-19 pandemic. BMC Res Notes 2020 October 21;13(1):493-6.

(36) Badahdah AM, Khamis F, Al Mahyijari N. Sleep quality among health care workers during the COVID-19 pandemic. J Clin Sleep Med 2020 September 15;16(9):1635.

(37) Zheng Y, Wang L, Feng L, Ye L, Zhang A, Fan R. Sleep quality and mental health of medical workers during the coronavirus disease 2019 pandemic. Sleep Biol Rhythms 2021 January 10:1-8.

(38) Tu ZH, He JW, Zhou N. Sleep quality and mood symptoms in conscripted frontline nurse in Wuhan, China during COVID-19 outbreak: A cross-sectional study. Medicine (Baltimore) 2020 June 26;99(26):e20769.

(39) Prasad A, Civantos AM, Byrnes Y, Chorath K, Poonia S, Chang C, et al. Snapshot Impact of COVID-19 on Mental Wellness in Nonphysician Otolaryngology Health Care Workers: A National Study. OTO Open 2020 August 07;4(3):2473974X20948835.

(40) Hennein R, Mew EJ, Lowe SR. Socio-ecological predictors of mental health outcomes among healthcare workers during the COVID-19 pandemic in the United States. PLoS One 2021 February 05;16(2):e0246602.

(41) Alan H, Eskin Bacaksiz F, Tiryaki Sen H, Taskiran Eskici G, Gumus E, Harmanci Seren AK. "I'm a hero, but...": An evaluation of depression, anxiety, and stress levels of frontline healthcare professionals during COVID-19 pandemic in Turkey. Perspect Psychiatr Care 2021 July 01;57(3):1126-1136.

(42) Hennein R, Lowe S. A hybrid inductive-abductive analysis of health workers' experiences and wellbeing during the COVID-19 pandemic in the United States. PLoS One 2020 October 26;15(10):e0240646.

(43) Chen R, Sun C, Chen JJ, Jen HJ, Kang XL, Kao CC, et al. A Large-Scale Survey on Trauma, Burnout, and Posttraumatic Growth among Nurses during the COVID-19 Pandemic. Int J Ment Health Nurs 2021 February 01;30(1):102-116.

(44) Chew NWS, Lee GKH, Tan BYQ, Jing M, Goh Y, Ngiam NJH, et al. A multinational, multicentre study on the psychological outcomes and associated physical symptoms amongst healthcare workers during COVID-19 outbreak. Brain Behav Immun 2020 August 01;88:559-565.

(45) Wang Y, Ma S, Yang C, Cai Z, Hu S, Zhang B, et al. Acute psychological effects of Coronavirus Disease 2019 outbreak among healthcare workers in China: a cross-sectional study. Transl Psychiatry 2020 October 13;10(1):348-w.

(46) Wang Y, Duan Z, Peng K, Li D, Ou J, Wilson A, et al. Acute Stress Disorder Among Frontline Health Professionals During the COVID-19 Outbreak: A Structural Equation Modeling Investigation. Psychosom Med 2021 May 01;83(4):373-379.

(47) Shahrour G, Dardas LA. Acute stress disorder, coping self-efficacy and subsequent psychological distress among nurses amid COVID-19. J Nurs Manag 2020 October 01;28(7):1686-1695.

(48) Fidanci I, Güleryüz OD, Fidanci I. AN analysis on sleep quality of the healthcare professionals during the COVID- 19 pandemic. Acta Medica Mediterranea 2020;36(6).

(49) Wu K, Wei X. Analysis of Psychological and Sleep Status and Exercise Rehabilitation of Front-Line Clinical Staff in the Fight Against COVID-19 in China. Med Sci Monit Basic Res 2020 May 11;26:e924085.

(50) Shen Y, Zhan Y, Zheng H, Liu H, Wan Y, Zhou W. Anxiety and its association with perceived stress and insomnia among nurses fighting against COVID-19 in Wuhan: A cross-sectional survey. J Clin Nurs 2021 September 01;30(17-18):2654-2664.

(51) Hasan SR, Hamid Z, Jawaid MT, Ali RK. Anxiety among Doctors during COVID-19 Pandemic in Secondary and Tertiary Care Hospitals. Pak J Med Sci 2020 October 01;36(6):1360-1365.

(52) Li W, Mao X, Li J, Fang L, Du G, Qiao J, et al. Anxiety and Depression Among Imaging Doctors in Post-COVID-19 Period. SN Compr Clin Med 2020 November 23:1-5.

(53) Pouralizadeh M, Bostani Z, Maroufizadeh S, Ghanbari A, Khoshbakht M, Alavi SA, et al. Anxiety and depression and the related factors in nurses of Guilan University of Medical Sciences hospitals during COVID-19: A web-based cross-sectional study. Int J Afr Nurs Sci 2020;13:100233.

(54) Xing LQ, Xu ML, Sun J, Wang QX, Ge DD, Jiang MM, et al. Anxiety and depression in frontline health care workers during the outbreak of Covid-19. Int J Soc Psychiatry 2020 October 24:20764020968119.

(55) Hassannia L, Taghizadeh F, Moosazadeh M, Zarghami M, Taghizadeh H, Dooki AF, et al. Anxiety and Depression in Health Workers and General Population During COVID-19 in IRAN: A Cross-Sectional Study. Neuropsychopharmacol Rep 2021 March 01;41(1):40-49.

(56) Han L, Wong FKY, She DLM, Li SY, Yang YF, Jiang MY, et al. Anxiety and Depression of Nurses in a North West Province in China During the Period of Novel Coronavirus Pneumonia Outbreak. J Nurs Scholarsh 2020 September 01;52(5):564-573.

(57) Gallopeni F, Bajraktari I, Selmani E, Tahirbegolli IA, Sahiti G, Muastafa A, et al. Anxiety and depressive symptoms among healthcare professionals during the Covid-19 pandemic in Kosovo: A cross sectional study. J Psychosom Res 2020 August 05;137:110212.

(58) Setiawati Y, Wahyuhadi J, Joestandari F, Maramis MM, Atika A. Anxiety and Resilience of Healthcare Workers During COVID-19 Pandemic in Indonesia. J Multidiscip Healthc 2021 January 06;14:1-8.

(59) Gupta B, Sharma V, Kumar N, Mahajan A. Anxiety and Sleep Disturbances Among Health Care Workers During the COVID-19 Pandemic in India: Cross-Sectional Online Survey. JMIR Public Health Surveill 2020 December 22;6(4):e24206.

(60) Cheng FF, Zhan SH, Xie AW, Cai SZ, Hui L, Kong XX, et al. Anxiety in Chinese pediatric medical staff during the outbreak of Coronavirus Disease 2019: a cross-sectional study. Transl Pediatr 2020 June 01;9(3):231-236.

(61) Yurtseven S, Arslan S. Anxiety levels of university hospital nurses during the Covid-19 pandemic. Perspect Psychiatr Care 2021 January 13.

(62) Wang QQ, Fang YY, Huang HL, Lv WJ, Wang XX, Yang TT, et al. Anxiety, depression and cognitive emotion regulation strategies in Chinese nurses during the COVID-19 outbreak. J Nurs Manag 2021 July 01;29(5):1263-1274.

(63) Di Tella M, Benfante A, Castelli L, Romeo A. Anxiety, depression, and posttraumatic stress in nurses during the COVID-19 outbreak. Intensive Crit Care Nurs 2021 June 01;64:103014.

(64) Awano N, Oyama N, Akiyama K, Inomata M, Kuse N, Tone M, et al. Anxiety, Depression, and Resilience of Healthcare Workers in Japan During the Coronavirus Disease 2019 Outbreak. Intern Med 2020;59(21):2693-2699.

(65) Chew NWS, Ngiam JN, Tan BY, Tham SM, Tan CY, Jing M, et al. Asian-Pacific perspective on the psychological well-being of healthcare workers during the evolution of the COVID-19 pandemic. BJPsych Open 2020 October 08;6(6):e116.

(66) Arshad MS, Hussain I, Nafees M, Majeed A, Imran I, Saeed H, et al. Assessing the Impact of COVID-19 on the Mental Health of Healthcare Workers in Three Metropolitan Cities of Pakistan. Psychol Res Behav Manag 2020 November 20;13:1047-1055.

(67) Wankowicz P, Szylinska A, Rotter I. Assessment of Mental Health Factors among Health Professionals Depending on Their Contact with COVID-19 Patients. Int J Environ Res Public Health 2020 August 12;17(16):10.3390/ijerph17165849.

(68) Lang Q, Liu X, He Y, Lv Q, Xu S. Association between working hours and anxiety/depression of medical staff during large-scale epidemic outbreak of COVID-19: A cross-sectional study. Psychiatry Investigation 2020;17(12).

(69) Macia-Rodriguez C, Alejandre de Ona A, Martin-Iglesias D, Barrera-Lopez L, Perez-Sanz MT, Moreno-Diaz J, et al. Burn-out syndrome in Spanish internists during the COVID-19 outbreak and associated factors: a cross-sectional survey. BMJ Open 2021 February 11;11(2):e042966-042966.

(70) Kamali M, Kalateh Sadati A, Khademi MR, Ghahramani S, Zarei L, Ghaemi SZ, et al. Burnout among Nurses during Coronavirus Disease 2019 Outbreak in Shiraz. Galen Med J 2020 December 26;9:e1956.

(71) Treluyer L, Tourneux P. Burnout among paediatric residents during the COVID-19 outbreak in France. Eur J Pediatr 2021 February 01;180(2):627-633.

(72) Duarte I, Teixeira A, Castro L, Marina S, Ribeiro C, Jácome C. Burnout among Portuguese healthcare workers during the COVID-19 pandemic. BMC Public Health 2020 Dec 7,;20(1).

(73) Chor WPD, Ng WM, Cheng L, Situ W, Chong JW, Ng LYA, et al. Burnout amongst emergency healthcare workers during the COVID-19 pandemic: A multi-center study. Am J Emerg Med 2021 August 01;46:700-702.

(74) Tan BYQ, Kanneganti A, Lim LJH, Tan M, Chua YX, Tan L, et al. Burnout and Associated Factors Among Health Care Workers in Singapore During the COVID-19 Pandemic. J Am Med Dir Assoc 2020 December 01;21(12):1751-1758.e5.

(75) Dobson H, Malpas CB, Burrell AJ, Gurvich C, Chen L, Kulkarni J, et al. Burnout and psychological distress amongst Australian healthcare workers during the COVID-19 pandemic. Australas Psychiatry 2021 February 01;29(1):26-30.

(76) Jose S, Dhandapani M, Cyriac MC. Burnout and Resilience among Frontline Nurses during COVID-19 Pandemic: A Cross-sectional Study in the Emergency Department of a Tertiary Care Center, North India. Indian J Crit Care Med 2020 November 01;24(11):1081-1088.

(77) Barello S, Palamenghi L, Graffigna G. Burnout and somatic symptoms among frontline healthcare professionals at the peak of the Italian COVID-19 pandemic. Psychiatry Res 2020 August 01;290:113129.

(78) Aydin Sayilan A, Kulakaç N, Uzun S. Burnout levels and sleep quality of COVID‐19 heroes. Perspectives in Psychiatric Care 2021 Jul 4,;57(3).

(79) Roslan NS, Yusoff MSB, Razak AA, Morgan K. Burnout Prevalence and Its Associated Factors among Malaysian Healthcare Workers during COVID-19 Pandemic: An Embedded Mixed-Method Study. Healthcare (Basel) 2021 January 17;9(1):10.3390/healthcare9010090.

(80) Podder I, Agarwal K, Datta S. Comparative analysis of perceived stress in dermatologists and other physicians during national lock-down and COVID-19 pandemic with exploration of possible risk factors: A web-based cross-sectional study from Eastern India. Dermatol Ther 2020 July 01;33(4):e13788.

(81) Milgrom Y, Tal Y, Finestone AS. Comparison of hospital worker anxiety in COVID-19 treating and non-treating hospitals in the same city during the COVID-19 pandemic. Isr J Health Policy Res 2020 October 21;9(1):55-1.

(82) Ahmed GK, Ramadan HK, Refay SM, Khashbah MA. Comparison of knowledge, attitude, socioeconomic burden, and mental health disorders of COVID-19 pandemic between general population and health care workers in Egypt. Egypt J Neurol Psychiatr Neurosurg 2021;57(1):25.

(83) Mora-Magana I, Lee SA, Maldonado-Castellanos I, Jimenez-Gutierrez C, Mendez-Venegas J, Maya-Del-Moral A, et al. Coronaphobia among healthcare professionals in Mexico: A psychometric analysis. Death Stud 2020 August 18:1-10.

(84) Altmayer V, Weiss N, Cao A, Marois C, Demeret S, Rohaut B, et al. Coronavirus disease 2019 crisis in Paris: A differential psychological impact between regular intensive care unit staff members and reinforcement workers. Aust Crit Care 2021 March 01;34(2):142-145.

(85) Arshad AR, Islam F. COVID-19 and Anxiety amongst Doctors: A Pakistani Perspective. J Coll Physicians Surg Pak 2020 October 01;30(10):106-109.

(86) Labrague LJ, De Los Santos, J A A. COVID-19 anxiety among front-line nurses: Predictive role of organisational support, personal resilience and social support. J Nurs Manag 2020 October 01;28(7):1653-1661.

(87) Guiroy A, Gagliardi M, Coombes N, Landriel F, Zanardi C, Willhuber GC, et al. COVID-19 Impact Among Spine Surgeons in Latin America. Global Spine J 2021 July 01;11(6):859-865.

(88) Liu X, Chen J, Wang D, Li X, Wang E, Jin Y, et al. COVID-19 Outbreak Can Change the Job Burnout in Health Care Professionals. Front Psychiatry 2020 December 08;11:563781.

(89) Naldi A, Vallelonga F, Di Liberto A, Cavallo R, Agnesone M, Gonella M, et al. COVID-19 pandemic-related anxiety, distress and burnout: prevalence and associated factors in healthcare workers of North-West Italy. BJPsych Open 2021 January 07;7(1):e27.

(90) Jain A, Singariya G, Kamal M, Kumar M, Jain A, Solanki RK. COVID-19 pandemic: Psychological impact on anaesthesiologists. Indian J Anaesth 2020 September 01;64(9):774-783.

(91) Saricam M. COVID-19-Related anxiety in nurses working on front lines in Turkey. Nursing and Midwifery Studies 2020;9(3).

(92) Mattila E, Peltokoski J, Neva MH, Kaunonen M, Helminen M, Parkkila AK. COVID-19: anxiety among hospital staff and associated factors. Ann Med 2021 December 01;53(1):237-246.

(93) Blekas A, Voitsidis P, Athanasiadou M, Parlapani E, Chatzigeorgiou AF, Skoupra M, et al. COVID-19: PTSD symptoms in Greek health care professionals. Psychol Trauma 2020 October 01;12(7):812-819.

(94) Jambunathan P, Jindal M, Patra P, Madhusudan T. COVID-warriors: Psychological impact of the severe acute respiratory syndrome coronavirus 2 pandemic on health-care professionals. Journal of Marine Medical Society 2020;0(0).

(95) Li L, Sun N, Fei S, Yu L, Chen S, Yang S, et al. Current status of and factors influencing anxiety and depression in front-line medical staff supporting Wuhan in containing the novel coronavirus pneumonia epidemic. Jpn J Nurs Sci 2021 April 01;18(2):e12398.

(96) Arafa A, Mohammed Z, Mahmoud O, Elshazley M, Ewis A. Depressed, anxious, and stressed: What have healthcare workers on the frontlines in Egypt and Saudi Arabia experienced during the COVID-19 pandemic? J Affect Disord 2021 January 01;278:365-371.

(97) Elbay RY, Kurtulmus A, Arpacioglu S, Karadere E. Depression, anxiety, stress levels of physicians and associated factors in Covid-19 pandemics. Psychiatry Res 2020 August 01;290:113130.

(98) Sunjaya DK, Herawati DMD, Siregar AYM. Depressive, anxiety, and burnout symptoms on health care personnel at a month after COVID-19 outbreak in Indonesia. BMC Public Health 2021 January 28;21(1):227-6.

(99) Salopek-Ziha D, Hlavati M, Gvozdanovic Z, Gasic M, Placento H, Jakic H, et al. Differences in Distress and Coping with the COVID-19 Stressor in Nurses and Physicians. Psychiatr Danub 2020 January 01;32(2):287-293.

(100) Mohd Fauzi MF, Mohd Yusoff H, Muhamad Robat R, Mat Saruan NA, Ismail KI, Mohd Haris AF. Doctors' Mental Health in the Midst of COVID-19 Pandemic: The Roles of Work Demands and Recovery Experiences. Int J Environ Res Public Health 2020 October 08;17(19):10.3390/ijerph17197340.

(101) Demartini B, Nistico V, D'Agostino A, Priori A, Gambini O. Early Psychiatric Impact of COVID-19 Pandemic on the General Population and Healthcare Workers in Italy: A Preliminary Study. Front Psychiatry 2020 December 22;11:561345.

(102) Erinoso O, Adejumo O, Fashina A, Falana A, Amure MT, Okediran OJ, et al. Effect of COVID-19 on mental health of frontline health workers in Nigeria: A preliminary cross-sectional study. J Psychosom Res 2020 December 01;139:110288.

(103) Wasim T, Raana G, Bushra N, Riaz A. Effect of COVID-19 Pandemic on Mental Wellbeing of Healthcare Workers in Tertiary Care Hospital. Annals of King Edward Medical University 2020;26(2).

(104) Marinaci T, Carpinelli L, Venuleo C, Savarese G, Cavallo P. Emotional distress, psychosomatic symptoms and their relationship with institutional responses: A survey of Italian frontline medical staff during the Covid-19 pandemic. Heliyon 2020 December 16;6(12):e05766.

(105) Thomaier L, Teoh D, Jewett P, Beckwith H, Parsons H, Yuan J, et al. Emotional health concerns of oncology physicians in the United States: Fallout during the COVID-19 pandemic. PLoS One 2020 November 24;15(11):e0242767.

(106) Thakre SS, Jadhao AR, Dhoble MA, Dass R, Thakre SB, Somani A. Evaluation of Effectiveness of COVID-19 Training and Assessment of Anxiety among Nurses of a Tertiary Health Care Centre during the Corona Virus Pandemic- An Experimental Study. JOURNAL OF CLINICAL AND DIAGNOSTIC RESEARCH 2020.

(107) Uyaroglu OA, Basaran NC, Ozisik L, Karahan S, Tanriover MD, Guven GS, et al. Evaluation of the effect of COVID-19 pandemic on anxiety severity of physicians working in the internal medicine department of a tertiary care hospital: a cross-sectional survey. Intern Med J 2020 November 01;50(11):1350-1358.

(108) Zhan Y, Liu Y, Liu H, Li M, Shen Y, Gui L, et al. Factors associated with insomnia among Chinese front-line nurses fighting against COVID-19 in Wuhan: A cross-sectional survey. J Nurs Manag 2020 October 01;28(7):1525-1535.

(109) Alshekaili M, Hassan W, Al Said N, Al Sulaimani F, Jayapal SK, Al-Mawali A, et al. Factors associated with mental health outcomes across healthcare settings in Oman during COVID-19: frontline versus non-frontline healthcare workers. BMJ Open 2020 October 10;10(10):e042030-042030.

(110) Lai J, Ma S, Wang Y, Cai Z, Hu J, Wei N, et al. Factors Associated With Mental Health Outcomes Among Health Care Workers Exposed to Coronavirus Disease 2019. JAMA Netw Open 2020 March 02;3(3):e203976.

(111) Gu Y, Zhu Y, Xu G. Factors associated with mental health outcomes among health care workers in the Fangcang shelter hospital in China. Int J Soc Psychiatry 2020 December 09:20764020975805.

(112) Khoodoruth MAS, Al-Nuaimi SK, Al-Salihy Z, Ghaffar A, Khoodoruth WNC, Ouanes S. Factors associated with mental health outcomes among medical residents exposed to COVID-19. BJPsych Open 2021 February 15;7(2):e52.

(113) Wang YX, Guo HT, Du XW, Song W, Lu C, Hao WN. Factors associated with post-traumatic stress disorder of nurses exposed to corona virus disease 2019 in China. Medicine (Baltimore) 2020 June 26;99(26):e20965.

(114) Jacob J, Vr V, Issac A, Stephen S, Dhandapani M, Vr R, et al. Factors associated with psychological outcomes among frontline healthcare providers of India during COVID-19 pandemic. Asian J Psychiatr 2021 January 01;55:102531.

(115) Li X, Zhou Y, Xu X. Factors associated with the psychological well-being among front-line nurses exposed to COVID-2019 in China: A predictive study. J Nurs Manag 2021 March 01;29(2):240-249.

(116) Conti C, Fontanesi L, Lanzara R, Rosa I, Porcelli P. Fragile heroes. The psychological impact of the COVID-19 pandemic on health-care workers in Italy. PLoS One 2020 November 18;15(11):e0242538.

(117) Di Monte C, Monaco S, Mariani R, Di Trani M. From Resilience to Burnout: Psychological Features of Italian General Practitioners During COVID-19 Emergency. Front Psychol 2020 October 02;11:567201.

(118) Hu D, Kong Y, Li W, Han Q, Zhang X, Zhu LX, et al. Frontline nurses' burnout, anxiety, depression, and fear statuses and their associated factors during the COVID-19 outbreak in Wuhan, China: A large-scale cross-sectional study. EClinicalMedicine 2020 June 27;24:100424.

(119) Teshome A, Glagn M, Shegaze M, Tekabe B, Getie A, Assefa G, et al. Generalized Anxiety Disorder and Its Associated Factors Among Health Care Workers Fighting COVID-19 in Southern Ethiopia. Psychol Res Behav Manag 2020 November 05;13:907-917.

(120) Bahadir-Yilmaz E, Yuksel A. State anxiety levels of nurses providing care to patients with COVID-19 in Turkey. Perspect Psychiatr Care 2021 July 01;57(3):1088-1094.

(121) Mosolova E, Chung S, Sosin D, Mosolov S. Stress and Anxiety among Healthcare Workers Associated with COVID-19 Pandemic in Russia. Psychiatr Danub 2020 January 01;32(3-4):549-556.

(122) Mathur S, Sharma D, Solanki R, Goyal M. Stress-related disorders in health-care workers in COVID-19 pandemic: A cross-sectional study from India. Indian Journal of Medical Specialities 2020;11(4):180.

(123) Xu X, Wang W, Chen J, Ai M, Shi L, Wang L, et al. Suicidal and self-harm ideation among Chinese hospital staff during the COVID-19 pandemic: Prevalence and correlates. Psychiatry Res 2021 February 01;296:113654.

(124) Zhang C, Yang L, Liu S, Ma S, Wang Y, Cai Z, et al. Survey of Insomnia and Related Social Psychological Factors Among Medical Staff Involved in the 2019 Novel Coronavirus Disease Outbreak. Front Psychiatry 2020 April 14;11:306.

(125) Sun Y, Geng W, He Y, Li Z, Jin M, Liu Y, et al. Survey of Mental Health Effects among Health Care Workers Involved with the COVID-19 Outbreak. Iran J Public Health 2020 November 01;49(11):2214-2216.

(126) Gupta S, Prasad AS, Dixit PK, Padmakumari P, Gupta S, Abhisheka K. Survey of prevalence of anxiety and depressive symptoms among 1124 healthcare workers during the coronavirus disease 2019 pandemic across India. Med J Armed Forces India 2021 July 01;77:S404-S412.

(127) Azoulay E, Cariou A, Bruneel F, Demoule A, Kouatchet A, Reuter D, et al. Symptoms of Anxiety, Depression, and Peritraumatic Dissociation in Critical Care Clinicians Managing Patients with COVID-19. A Cross-Sectional Study. Am J Respir Crit Care Med 2020 November 15;202(10):1388-1398.

(128) Luceno-Moreno L, Talavera-Velasco B, Garcia-Albuerne Y, Martin-Garcia J. Symptoms of Posttraumatic Stress, Anxiety, Depression, Levels of Resilience and Burnout in Spanish Health Personnel during the COVID-19 Pandemic. Int J Environ Res Public Health 2020 July 30;17(15):10.3390/ijerph17155514.

(129) Young KP, Kolcz DL, O'Sullivan DM, Ferrand J, Fried J, Robinson K. Health Care Workers' Mental Health and Quality of Life During COVID-19: Results From a Mid-Pandemic, National Survey. Psychiatr Serv 2021 February 01;72(2):122-128.

(130) Korkmaz S, Kazgan A, Cekic S, Tartar AS, Balci HN, Atmaca M. The anxiety levels, quality of sleep and life and problem-solving skills in healthcare workers employed in COVID-19 services. J Clin Neurosci 2020 October 01;80:131-136.

(131) Mosheva M, Gross R, Hertz-Palmor N, Hasson-Ohayon I, Kaplan R, Cleper R, et al. The association between witnessing patient death and mental health outcomes in frontline COVID-19 healthcare workers. Depress Anxiety 2021 April 01;38(4):468-479.

(132) Rapisarda F, Vallarino M, Cavallini E, Barbato A, Brousseau-Paradis C, De Benedictis L, et al. The Early Impact of the Covid-19 Emergency on Mental Health Workers: A Survey in Lombardy, Italy. Int J Environ Res Public Health 2020 November 20;17(22):10.3390/ijerph17228615.

(133) Crowe S, Howard AF, Vanderspank-Wright B, Gillis P, McLeod F, Penner C, et al. The effect of COVID-19 pandemic on the mental health of Canadian critical care nurses providing patient care during the early phase pandemic: A mixed method study. Intensive Crit Care Nurs 2021 April 01;63:102999.

(134) Yas S, Bildik F, Aslaner M, Aslan S, Keles A, Kilicaslan I et al. The Effect of the COVID-19 Pandemic on the Psychological Status of Hospital Workers. Psychiatry and Clinical Psychopharmacology 2020;30(3):1.

(135) Çaliskan F. The Evaluation of Knowledge, Attitudes, Depression and Anxiety Levels among Emergency Physicians during the COVID-19 Pandemic. Signa Vitae 2020;16(1):163.

(136) Qi J, Xu J, Li BZ, Huang JS, Yang Y, Zhang ZT, et al. The evaluation of sleep disturbances for Chinese frontline medical workers under the outbreak of COVID-19. Sleep Med 2020 August 01;72:1-4.

(137) Jahrami H, BaHammam AS, AlGahtani H, Ebrahim A, Faris M, AlEid K, et al. The examination of sleep quality for frontline healthcare workers during the outbreak of COVID-19. Sleep Breath 2021 March 01;25(1):503-511.

(138) Karasu F, Ozturk Copur E, Ayar D. The impact of COVID-19 on healthcare workers' anxiety levels. Z Gesundh Wiss 2021 January 04:1-11.

(139) Geng S, Zhou Y, Zhang W, Lou A, Cai Y, Xie J, et al. The influence of risk perception for COVID-19 pandemic on posttraumatic stress disorder in healthcare workers: A survey from four designated hospitals. Clin Psychol Psychother 2021 February 16.

(140) Lin K, Yang BX, Luo D, Liu Q, Ma S, Huang R, et al. The Mental Health Effects of COVID-19 on Health Care Providers in China. Am J Psychiatry 2020 July 01;177(7):635-636.

(141) Khamis F, Al Mahyijari N, Al Lawati F, Badahdah AM. The Mental Health of Female Physicians and Nurses in Oman during the COVID-19 Pandemic. Oman Med J 2020 November 30;35(6):e203.

(142) Cai Q, Feng H, Huang J, Wang M, Wang Q, Lu X, et al. The mental health of frontline and non-frontline medical workers during the coronavirus disease 2019 (COVID-19) outbreak in China: A case-control study. J Affect Disord 2020 October 01;275:210-215.

(143) Badahdah A, Khamis F, Al Mahyijari N, Al Balushi M, Al Hatmi H, Al Salmi I, et al. The mental health of health care workers in Oman during the COVID-19 pandemic. Int J Soc Psychiatry 2021 February 01;67(1):90-95.

(144) Ning X, Yu F, Huang Q, Li X, Luo Y, Huang Q, et al. The mental health of neurological doctors and nurses in Hunan Province, China during the initial stages of the COVID-19 outbreak. BMC Psychiatry 2020 September 05;20(1):436-z.

(145) Ide K, Asami T, Suda A, Yoshimi A, Fujita J, Nomoto M et al. The psychological effects of COVID-19 on hospital workers at the beginning of the outbreak with a large disease cluster on the Diamond Princess cruise ship. PLOS ONE 2020;16(1):e0245294.

(146) Li Q, Chen J, Xu G, Zhao J, Yu X, Wang S, et al. The Psychological Health Status of Healthcare Workers During the COVID-19 Outbreak: A Cross-Sectional Survey Study in Guangdong, China. Front Public Health 2020 September 18;8:562885.

(147) Saracoglu KT, Simsek T, Kahraman S, Bombaci E, Sezen O, Saracoglu A, et al. The Psychological Impact of COVID-19 Disease is more Severe on Intensive Care Unit Healthcare Providers: A Cross-sectional Study. Clin Psychopharmacol Neurosci 2020 November 30;18(4):607-615.

(148) Teo WZY, Yap ES, Yip C, Ong L, Lee CT. The psychological impact of COVID-19 on 'hidden' frontline healthcare workers. Int J Soc Psychiatry 2021 May 01;67(3):284-289.

(149) Castelli L, Di Tella M, Benfante A, Taraschi A, Bonagura G, Pizzini A, et al. The psychological impact of COVID-19 on general practitioners in Piedmont, Italy. J Affect Disord 2021 February 15;281:244-246.

(150) Chen B, Li QX, Zhang H, Zhu JY, Yang X, Wu YH, et al. The psychological impact of COVID-19 outbreak on medical staff and the general public. Curr Psychol 2020 October 07:1-9.

(151) Mekonen E, Shetie B, Muluneh N. The Psychological Impact of COVID-19 Outbreak on Nurses Working in the Northwest of Amhara Regional State Referral Hospitals, Northwest Ethiopia. Psychol Res Behav Manag 2021 January 05;13:1353-1364.

(152) Wang H, Huang D, Huang H, Zhang J, Guo L, Liu Y, et al. The psychological impact of COVID-19 pandemic on medical staff in Guangdong, China: a cross-sectional study. Psychol Med 2020 July 06:1-9.

(153) Giusti EM, Pedroli E, D'Aniello GE, Stramba Badiale C, Pietrabissa G, Manna C, et al. The Psychological Impact of the COVID-19 Outbreak on Health Professionals: A Cross-Sectional Study. Front Psychol 2020 July 10;11:1684.

(154) Al Mahyijari N, Badahdah A, Khamis F. The psychological impacts of COVID-19: a study of frontline physicians and nurses in the Arab world. Ir J Psychol Med 2021 September 01;38(3):186-191.

(155) Xiong H, Yi S, Lin Y. The Psychological Status and Self-Efficacy of Nurses During COVID-19 Outbreak: A Cross-Sectional Survey. Inquiry 2020 December 01;57:46958020957114.

(156) Xiaoming X, Ming A, Su H, Wo W, Jianmei C, Qi Z, et al. The psychological status of 8817 hospital workers during COVID-19 Epidemic: A cross-sectional study in Chongqing. J Affect Disord 2020 November 01;276:555-561.

(157) Li H, Zhang Y, Wang H, Liang J, Zhou Y, Huang Y, et al. The Relationship Between Symptoms of Anxiety and Somatic Symptoms in Health Professionals During the Coronavirus Disease 2019 Pandemic. Neuropsychiatr Dis Treat 2020 December 18;16:3153-3161.

(158) Xia Y, Zhang H, Xia Y, Li H, Zhai L, Wang H. The self-psychological safety maintenance and its influencing factors of community frontline staff during COVID-19 pandemic. Medicine (Baltimore) 2021 January 22;100(3):e24140.

(159) Teshome A, Glagn M, Shegaze M, Tekabe B, Getie A, Assefa G, et al. Generalized Anxiety Disorder and Its Associated Factors Among Health Care Workers Fighting COVID-19 in Southern Ethiopia. Psychol Res Behav Manag 2020 November 05;13:907-917.

(160) Young KP, Kolcz DL, O'Sullivan DM, Ferrand J, Fried J, Robinson K. Health Care Workers' Mental Health and Quality of Life During COVID-19: Results From a Mid-Pandemic, National Survey. Psychiatr Serv 2021 February 01;72(2):122-128.

(161) Amra B, Salmasi M, Soltaninejad F, Sami R, Nickpour M, Mansourian M, et al. Healthcare workers' sleep and mood disturbances during COVID-19 outbreak in an Iranian referral center. Sleep Breath 2021 February 13.

(162) Halayem S, Sayari N, Cherif W, Cheour M, Damak R. How Tunisian physicians of public health hospitals deal with COVID-19 pandemic: Perceived stress and coping strategies. Psychiatry Clin Neurosci 2020 September 01;74(9):496-497.

(163) Hong S, Ai M, Xu X, Wang W, Chen J, Zhang Q, et al. Immediate psychological impact on nurses working at 42 government-designated hospitals during COVID-19 outbreak in China: A cross-sectional study. Nurs Outlook 2021 February 01;69(1):6-12.

(164) Cui S, Jiang Y, Shi Q, Zhang L, Kong D, Qian M, et al. Impact of COVID-19 on Anxiety, Stress, and Coping Styles in Nurses in Emergency Departments and Fever Clinics: A Cross-Sectional Survey. Risk Manag Healthc Policy 2021 February 15;14:585-594.

(165) Sandesh R, Shahid W, Dev K, Mandhan N, Shankar P, Shaikh A, et al. Impact of COVID-19 on the Mental Health of Healthcare Professionals in Pakistan. Cureus 2020 July 02;12(7):e8974.

(166) Chatzittofis A, Karanikola M, Michailidou K, Constantinidou A. Impact of the COVID-19 Pandemic on the Mental Health of Healthcare Workers. Int J Environ Res Public Health 2021 February 03;18(4):10.3390/ijerph18041435.

(167) Kang L, Ma S, Chen M, Yang J, Wang Y, Li R, et al. Impact on mental health and perceptions of psychological care among medical and nursing staff in Wuhan during the 2019 novel coronavirus disease outbreak: A cross-sectional study. Brain Behav Immun 2020 July 01;87:11-17.

(168) Tran TV, Nguyen HC, Pham LV, Nguyen MH, Nguyen HC, Ha TH, et al. Impacts and interactions of COVID-19 response involvement, health-related behaviours, health literacy on anxiety, depression and health-related quality of life among healthcare workers: a cross-sectional study. BMJ Open 2020 December 07;10(12):e041394-041394.

(169) Zhang C, Peng D, Lv L, Zhuo K, Yu K, Shen T, et al. Individual Perceived Stress Mediates Psychological Distress in Medical Workers During COVID-19 Epidemic Outbreak in Wuhan. Neuropsychiatr Dis Treat 2020 October 28;16:2529-2537.

(170) Sagherian K, Steege LM, Cobb SJ, Cho H. Insomnia, fatigue and psychosocial well-being during COVID-19 pandemic: A cross-sectional survey of hospital nursing staff in the United States. J Clin Nurs 2020 November 20.

(171) Tselebis A, Lekka D, Sikaras C, Tsomaka E, Tassopoulos A, Ilias I, et al. Insomnia, Perceived Stress, and Family Support among Nursing Staff during the Pandemic Crisis. Healthcare (Basel) 2020 October 26;8(4):10.3390/healthcare8040434.

(172) Shen M, Xu H, Fu J, Wang T, Fu Z, Zhao X, et al. Investigation of anxiety levels of 1637 healthcare workers during the epidemic of COVID-19. PLoS One 2020 December 22;15(12):e0243890.

(173) An Y, Sun Y, Liu Z, Chen Y. Investigation of the mental health status of frontier-line and non-frontier-line medical staff during a stress period. J Affect Disord 2021 March 01;282:836-839.

(174) Zhao X, Zhang T, Li B, Yu X, Ma Z, Cao L, et al. Job-related factors associated with changes in sleep quality among healthcare workers screening for 2019 novel coronavirus infection: a longitudinal study. Sleep Med 2020 November 01;75:21-26.

(175) Jindal V, Mittal S, Kaur T, Bansal AS, Kaur P, Kaur G, et al. Knowledge, anxiety and the use of hydroxychloroquine prophylaxis among health care students and professionals regarding COVID-19 pandemic. Adv Respir Med 2020;88(6):520-530.

(176) Lasalvia A, Amaddeo F, Porru S, Carta A, Tardivo S, Bovo C, et al. Levels of burn-out among healthcare workers during the COVID-19 pandemic and their associated factors: a cross-sectional study in a tertiary hospital of a highly burdened area of north-east Italy. BMJ Open 2021 January 17;11(1):e045127-045127.

(177) Roberts NJ, McAloney-Kocaman K, Lippiett K, Ray E, Welch L, Kelly C. Levels of resilience, anxiety and depression in nurses working in respiratory clinical areas during the COVID pandemic. Respir Med 2021 January 01;176:106219.

(178) Leng M, Wei L, Shi X, Cao G, Wei Y, Xu H, et al. Mental distress and influencing factors in nurses caring for patients with COVID-19. Nurs Crit Care 2021 March 01;26(2):94-101.

(179) Civantos AM, Bertelli A, Goncalves A, Getzen E, Chang C, Long Q, et al. Mental health among head and neck surgeons in Brazil during the COVID-19 pandemic: A national study. Am J Otolaryngol 2020 December 01;41(6):102694.

(180) AlAteeq DA, Aljhani S, Althiyabi I, Majzoub S. Mental health among healthcare providers during coronavirus disease (COVID-19) outbreak in Saudi Arabia. J Infect Public Health 2020 October 01;13(10):1432-1437.

(181) Hummel S, Oetjen N, Du J, Posenato E, Resende de Almeida, R M, Losada R, et al. Mental Health Among Medical Professionals During the COVID-19 Pandemic in Eight European Countries: Cross-sectional Survey Study. J Med Internet Res 2021 January 18;23(1):e24983.

(182) Manh Than H, Minh Nong V, Trung Nguyen C, Phu Dong K, Ngo HT, Thu Doan T, et al. Mental Health and Health-Related Quality-of-Life Outcomes Among Frontline Health Workers During the Peak of COVID-19 Outbreak in Vietnam: A Cross-Sectional Study. Risk Manag Healthc Policy 2020 December 08;13:2927-2936.

(183) Suryavanshi N, Kadam A, Dhumal G, Nimkar S, Mave V, Gupta A, et al. Mental health and quality of life among healthcare professionals during the COVID-19 pandemic in India. Brain Behav 2020 November 01;10(11):e01837.

(184) Gorini A, Fiabane E, Sommaruga M, Barbieri S, Sottotetti F, La Rovere MT, et al. Mental health and risk perception among Italian healthcare workers during the second month of the Covid-19 pandemic. Arch Psychiatr Nurs 2020 December 01;34(6):537-544.

(185) Tian T, Meng F, Pan W, Zhang S, Cheung T, Ng CH, et al. Mental health burden of frontline health professionals treating imported patients with COVID-19 in China during the pandemic. Psychol Med 2020 May 29:1-2.

(186) He Q, Fan B, Xie B, Liao Y, Han X, Chen Y, et al. Mental health conditions among the general population, healthcare workers and quarantined population during the coronavirus disease 2019 (COVID-19) pandemic. Psychol Health Med 2020 December 30:1-13.

(187) Alonso J, Vilagut G, Mortier P, Ferrer M, Alayo I, Aragon-Pena A, et al. Mental health impact of the first wave of COVID-19 pandemic on Spanish healthcare workers: A large cross-sectional survey. Rev Psiquiatr Salud Ment (Engl Ed) 2021 June 01;14(2):90-105.

(188) Khanal P, Devkota N, Dahal M, Paudel K, Joshi D. Mental health impacts among health workers during COVID-19 in a low resource setting: a cross-sectional survey from Nepal. Global Health 2020 September 25;16(1):89-z.

(189) Liang Y, Wu K, Zhou Y, Huang X, Zhou Y, Liu Z. Mental Health in Frontline Medical Workers during the 2019 Novel Coronavirus Disease Epidemic in China: A Comparison with the General Population. Int J Environ Res Public Health 2020 September 09;17(18):10.3390/ijerph17186550.

(190) Elkholy H, Tawfik F, Ibrahim I, Salah El-Din W, Sabry M, Mohammed S, et al. Mental health of frontline healthcare workers exposed to COVID-19 in Egypt: A call for action. Int J Soc Psychiatry 2021 August 01;67(5):522-531.

(191) Yitayih Y, Mekonen S, Zeynudin A, Mengistie E, Ambelu A. Mental health of healthcare professionals during the early stage of the COVID-19 pandemic in Ethiopia. BJPsych Open 2020 December 01;7(1):e1.

(192) Antonijevic J, Binic I, Zikic O, Manojlovic S, Tosic-Golubovic S, Popovic N. Mental health of medical personnel during the COVID-19 pandemic. Brain Behav 2020 December 01;10(12):e01881.

(193) Kounou KB, Guedenon KM, Dogbe Foli AA, Gnassounou-Akpa E. Mental health of medical professionals during the COVID-19 pandemic in Togo. Psychiatry Clin Neurosci 2020 October 01;74(10):559-560.

(194) Eduardo Bassani Dal'Bosco, Lara Simone Messias Floriano, Suellen Vienscoski Skupien, Guilherme Arcaro, Alessandra Rodrigues Martins, Aline Cristina Correa Anselmo. Mental health of nursing in coping with COVID-19 at a regional university hospital. Rev Bras Enferm 2020;73(2):e20200434.

(195) Jokic-Begic N, Lauri Korajlija A, Begic D. Mental Health of Psychiatrists and Physicians of Other Specialties in Early COVID-19 Pandemic: Risk ind Protective Factors. Psychiatr Danub 2020 January 01;32(3-4):536-548.

(196) Greenberg N, Weston D, Hall C, Caulfield T, Williamson V, Fong K. Mental health of staff working in intensive care during Covid-19. Occup Med (Lond) 2021 April 09;71(2):62-67.

(197) Rossi R, Socci V, Pacitti F, Di Lorenzo G, Di Marco A, Siracusano A, et al. Mental Health Outcomes Among Frontline and Second-Line Health Care Workers During the Coronavirus Disease 2019 (COVID-19) Pandemic in Italy. JAMA Netw Open 2020 May 01;3(5):e2010185.

(198) Rossi R, Socci V, Pacitti F, Mensi S, Di Marco A, Siracusano A, et al. Mental Health Outcomes Among Healthcare Workers and the General Population During the COVID-19 in Italy. Front Psychol 2020 December 08;11:608986.

(199) Al Ammari M, Sultana K, Thomas A, Al Swaidan L, Al Harthi N. Mental Health Outcomes Amongst Health Care Workers During COVID 19 Pandemic in Saudi Arabia. Front Psychiatry 2021 January 14;11:619540.

(200) Tiete J, Guatteri M, Lachaux A, Matossian A, Hougardy JM, Loas G, et al. Mental Health Outcomes in Healthcare Workers in COVID-19 and Non-COVID-19 Care Units: A Cross-Sectional Survey in Belgium. Front Psychol 2021 January 05;11:612241.

(201) Robles R, Rodriguez E, Vega-Ramirez H, Alvarez-Icaza D, Madrigal E, Durand S, et al. Mental health problems among healthcare workers involved with the COVID-19 outbreak. Braz J Psychiatry 2020 December 18.

(202) Szepietowski JC, Krajewski P, Bilynicki-Birula R, Poznanski P, Krajewska M, Rymaszewska J, et al. Mental health status of health care workers during the COVID-19 outbreak in Poland: One region, two different settings. Dermatol Ther 2020 November 01;33(6):e13855.

(203) Youssef N, Mostafa A, Ezzat R, Yosef M, El Kassas M. Mental health status of health-care professionals working in quarantine and non-quarantine Egyptian hospitals during the COVID-19 pandemic. East Mediterr Health J 2020 October 13;26(10):1155-1164.

(204) Song X, Fu W, Liu X, Luo Z, Wang R, Zhou N, et al. Mental health status of medical staff in emergency departments during the Coronavirus disease 2019 epidemic in China. Brain Behav Immun 2020 August 01;88:60-65.

(205) Liu Y, Wang L, Chen L, Zhang X, Bao L, Shi Y. Mental Health Status of Paediatric Medical Workers in China During the COVID-19 Outbreak. Front Psychiatry 2020 July 21;11:702.

(206) Naser AY, Dahmash EZ, Al-Rousan R, Alwafi H, Alrawashdeh HM, Ghoul I, et al. Mental health status of the general population, healthcare professionals, and university students during 2019 coronavirus disease outbreak in Jordan: A cross-sectional study. Brain Behav 2020 August 01;10(8):e01730.

(207) Wanigasooriya K, Palimar P, Naumann DN, Ismail K, Fellows JL, Logan P, et al. Mental health symptoms in a cohort of hospital healthcare workers following the first peak of the COVID-19 pandemic in the UK. BJPsych Open 2020 December 29;7(1):e24.

(208) Huang L, Lei W, Liu H, Hang R, Tao X, Zhan Y. Nurses' Sleep Quality of "Fangcang" Hospital in China during the COVID-19 Pandemic. Int J Ment Health Addict 2020 October 26:1-11.

(209) Havaei F, Ma A, Staempfli S, MacPhee M. Nurses' Workplace Conditions Impacting Their Mental Health during COVID-19: A Cross-Sectional Survey Study. Healthcare (Basel) 2021 January 16;9(1):10.3390/healthcare9010084.

(210) Vafaei H, Roozmeh S, Hessami K, Kasraeian M, Asadi N, Faraji A, et al. Obstetrics Healthcare Providers' Mental Health and Quality of Life During COVID-19 Pandemic: Multicenter Study from Eight Cities in Iran. Psychol Res Behav Manag 2020 July 17;13:563-571.

(211) Zhang X, Zhao K, Zhang G, Feng R, Chen J, Xu D, et al. Occupational Stress and Mental Health: A Comparison Between Frontline Medical Staff and Non-frontline Medical Staff During the 2019 Novel Coronavirus Disease Outbreak. Front Psychiatry 2020 December 23;11:555703.

(212) Sun Y, Song H, Liu H, Mao F, Sun X, Cao F. Occupational stress, mental health, and self-efficacy among community mental health workers: A cross-sectional study during COVID-19 pandemic. Int J Soc Psychiatry 2020 November 11:20764020972131.

(213) Wright HM, Griffin BJ, Shoji K, Love TM, Langenecker SA, Benight CC, et al. Pandemic-related mental health risk among front line personnel. J Psychiatr Res 2021 May 01;137:673-680.

(214) Lam SC, Arora T, Grey I, Suen LKP, Huang EY, Li D, et al. Perceived Risk and Protection From Infection and Depressive Symptoms Among Healthcare Workers in Mainland China and Hong Kong During COVID-19. Front Psychiatry 2020 July 15;11:686.

(215) Arnetz JE, Goetz CM, Sudan S, Arble E, Janisse J, Arnetz BB. Personal Protective Equipment and Mental Health Symptoms Among Nurses During the COVID-19 Pandemic. J Occup Environ Med 2020 November 01;62(11):892-897.

(216) Haravuori H, Junttila K, Haapa T, Tuisku K, Kujala A, Rosenstrom T, et al. Personnel Well-Being in the Helsinki University Hospital during the COVID-19 Pandemic-A Prospective Cohort Study. Int J Environ Res Public Health 2020 October 28;17(21):10.3390/ijerph17217905.

(217) Marco CA, Larkin GL, Feeser VR, Monti JE, Vearrier L, ACEP Ethics Committee. Post-traumatic stress and stress disorders during the COVID-19 pandemic: Survey of emergency physicians. J Am Coll Emerg Physicians Open 2020 November 02;1(6):1594-1601.

(218) Zhang H, Shi Y, Jing P, Zhan P, Fang Y, Wang F. Posttraumatic stress disorder symptoms in healthcare workers after the peak of the COVID-19 outbreak: A survey of a large tertiary care hospital in Wuhan. Psychiatry Res 2020 December 01;294:113541.

(219) Yin Q, Sun Z, Liu T, Ni X, Deng X, Jia Y, et al. Posttraumatic stress symptoms of health care workers during the corona virus disease 2019. Clin Psychol Psychother 2020 May 01;27(3):384-395.

(220) Pang Y, Fang H, Li L, Chen M, Chen Y, Chen M. Predictive factors of anxiety and depression among nurses fighting coronavirus disease 2019 in China. Int J Ment Health Nurs 2021 April 01;30(2):524-532.

(221) Kim SC, Quiban C, Sloan C, Montejano A. Predictors of poor mental health among nurses during COVID-19 pandemic. Nurs Open 2021 March 01;8(2):900-907.

(222) Zheng R, Zhou Y, Qiu M, Yan Y, Yue J, Yu L, et al. Prevalence and associated factors of depression, anxiety, and stress among Hubei pediatric nurses during COVID-19 pandemic. Compr Psychiatry 2021 January 01;104:152217.

(223) Zhou Y, Yang Y, Shi T, Song Y, Zhou Y, Zhang Z, et al. Prevalence and Demographic Correlates of Poor Sleep Quality Among Frontline Health Professionals in Liaoning Province, China During the COVID-19 Outbreak. Front Psychiatry 2020 June 12;11:520.

(224) Zhu J, Sun L, Zhang L, Wang H, Fan A, Yang B, et al. Prevalence and Influencing Factors of Anxiety and Depression Symptoms in the First-Line Medical Staff Fighting Against COVID-19 in Gansu. Front Psychiatry 2020 April 29;11:386.

(225) Zhan YX, Zhao SY, Yuan J, Liu H, Liu YF, Gui LL, et al. Prevalence and Influencing Factors on Fatigue of First-line Nurses Combating with COVID-19 in China: A Descriptive Cross-Sectional Study. Curr Med Sci 2020 August 01;40(4):625-635.

(226) Labrague LJ, De Los Santos, J A A. Prevalence and predictors of coronaphobia among frontline hospital and public health nurses. Public Health Nurs 2021 May 01;38(3):382-389.

(227) Vallee M, Kutchukian S, Pradere B, Verdier E, Durbant E, Ramlugun D, et al. Prospective and observational study of COVID-19's impact on mental health and training of young surgeons in France. Br J Surg 2020 October 01;107(11):e486-e488.

(228) Yu X, Li Y, Tang L, Deng L, Zhao Y, Zhao X, et al. Psychological Behavior of Frontline Medical Staff in the Use of Preventive Medication for COVID-19: A Cross-Sectional Study. Front Psychol 2020 September 25;11:2104.

(229) Skoda EM, Teufel M, Stang A, Jockel KH, Junne F, Weismuller B, et al. Psychological burden of healthcare professionals in Germany during the acute phase of the COVID-19 pandemic: differences and similarities in the international context. J Public Health (Oxf) 2020 November 23;42(4):688-695.

(230) Nguepy Keubo FR, Mboua PC, Djifack Tadongfack T, Fokouong Tchoffo E, Tasson Tatang C, Ide Zeuna J, et al. Psychological distress among health care professionals of the three COVID-19 most affected Regions in Cameroon: Prevalence and associated factors. Ann Med Psychol (Paris) 2021 February 01;179(2):141-146.

(231) Park SY, Kim B, Jung DS, Jung SI, Oh WS, Kim SW, et al. Psychological distress among infectious disease physicians during the response to the COVID-19 outbreak in the Republic of Korea. BMC Public Health 2020 November 27;20(1):1811-w.

(232) Lee MCC, Thampi S, Chan HP, Khoo D, Chin BZB, Foo DPX, et al. Psychological distress during the COVID-19 pandemic amongst anaesthesiologists and nurses. Br J Anaesth 2020 October 01;125(4):e384-e386.

(233) Bizri M, Kassir G, Tamim H, Kobeissy F, Hayek SE. Psychological distress experienced by physicians and nurses at a tertiary care center in Lebanon during the COVID-19 outbreak. J Health Psychol 2021 February 10:1359105321991630.

(234) Juan Y, Yuanyuan C, Qiuxiang Y, Cong L, Xiaofeng L, Yundong Z, et al. Psychological distress surveillance and related impact analysis of hospital staff during the COVID-19 epidemic in Chongqing, China. Compr Psychiatry 2020 November 01;103:152198.

(235) Gupta S, Kohli K, Padmakumari P, Dixit PK, Prasad AS, Chakravarthy BS, et al. Psychological Health Among Armed Forces Doctors During COVID-19 Pandemic in India. Indian J Psychol Med 2020 July 14;42(4):374-378.

(236) Tan YQ, Wang Z, Yap QV, Chan YH, Ho RC, Hamid, A R A H, et al. Psychological Health of Surgeons in a Time of COVID-19: A Global Survey. Ann Surg 2021 January 22.

stylefix
